# Shared and unique metabolic features of the malignant and benign thyroid lesions determined with use of ^1^H HR MAS NMR spectroscopy

**DOI:** 10.1038/s41598-020-79565-8

**Published:** 2021-01-14

**Authors:** Agnieszka Skorupa, Mateusz Ciszek, Ewa Chmielik, Łukasz Boguszewicz, Małgorzata Oczko-Wojciechowska, Małgorzata Kowalska, Dagmara Rusinek, Tomasz Tyszkiewicz, Aneta Kluczewska-Gałka, Agnieszka Czarniecka, Barbara Jarząb, Maria Sokół

**Affiliations:** 1 Department of Medical Physics, Maria Skłodowska-Curie National Research Institute of Oncology, Gliwice Branch, 44‐102 Gliwice, Poland; 2 Tumor Pathology Department, Maria Skłodowska-Curie National Research Institute of Oncology, Gliwice Branch, 44‐102 Gliwice, Poland; 3 Department of Genetic and Molecular Diagnostics of Cancer, Maria Skłodowska-Curie National Research Institute of Oncology, Gliwice Branch, 44‐102 Gliwice, Poland; 4 Department of Nuclear Medicine and Endocrine Oncology, Maria Skłodowska-Curie National Research Institute of Oncology, Gliwice Branch, 44‐102 Gliwice, Poland; 5 The Oncologic and Reconstructive Surgery Clinic, Maria Skłodowska-Curie National Research Institute of Oncology, Gliwice Branch, 44‐102 Gliwice, Poland

**Keywords:** Tumour biomarkers, Cancer metabolism

## Abstract

The purpose of this work was to investigate the distinct and common metabolic features of the malignant and benign thyroid lesions in reference to the non-transformed tissue from the contralateral gland (chronic thyroiditis and colloid goiter). ^1^H HR MAS NMR spectra of 38 malignant lesions, 32 benign lesions and 112 samples from the non-tumoral tissue (32 from chronic thyroiditis and 80 samples from colloid goiter) were subjected both to multivariate and univariate analysis. The increased succinate, glutamine, glutathione, serine/cysteine, ascorbate, lactate, taurine, threonine, glycine, phosphocholine/glycerophosphocholine and decreased lipids were found in both lesion types in comparison to either colloid goiter or chronic thyroiditis. The elevated glutamate and choline, and reduced citrate and glucose were additionally evident in these lesions in reference to goiter, while the increased myo-inositol—in comparison to thyroiditis. The malignant lesions were characterized by the higher alanine and lysine levels than colloid goiter and thyroiditis, while scyllo-inositol was uniquely increased in the benign lesions (not in cancer) in comparison to both non-tumoral tissue types. Moreover, the benign lesions presented with the unique increase of choline in reference to thyroiditis (not observed in the cancerous tissue). The metabolic heterogeneity of the non-tumoral tissue should be considered in the analysis of metabolic reprogramming in the thyroid lesions.

## Introduction

Thyroid nodules are defined as discrete lesions within the thyroid gland. They are very common in general population: physical examination allows their detection in 4–7% adults, while their prevalence is estimated at 19–67% using ultrasonography^1–3^. The majority of the nodules are benign and require a conservative management. However, from 4.0% to 6.5% of the palpable lesions represent thyroid cancer^4,5^. The World Health Organization data^6^ and the Cancer Incidence in Five Continents reports^7^ reveal an upward trend in incidence and a downward trend in mortality in thyroid cancer, though varying widely by country. A steady increase in the incidence of thyroid cancer (mainly papillary carcinomas accounting for approximately 85% of thyroid cancers) observed in both sexes in most countries is the most likely due to a substantial increment in the detection of subclinical thyroid cancers over the last decades, particularly through the use of ultrasounds, ultrasound-guided fine-needle aspiration and computed tomography. This trend, largely or totally, reflects overdiagnosis of indolent disease, i.e., small papillary carcinomas. In case of more rare histotypes of thyroid cancer, like follicular, medullary and anaplastic thyroid cancers, the management and prognosis are substantially less favorable than for the papillary ones^4^.

In order to support the preoperative diagnosis a better understanding of the molecular mechanisms underlying thyroid cancer biology are required. Various -omics technologies are intensively exploited for this purpose^8^. Metabolomics—one of the latest -omics platforms, deals with the study of metabolic reprogramming dictated by the cancer cells and performed to cope with unfavorable conditions in the tumor microenvironment (low oxygen tension and nutrients deficiency). Some of the most striking changes of tumor cellular bioenergetics, like enhanced aerobic glycolysis, glutaminolysis, altered TCA cycle and fatty acids metabolism in thyroid cancer have already been proved using NMR and mass spectrometry^9–16^. Since both benign and malignant thyroid lesions contain proliferating cells, some of the biochemical processes leading to the biomass increase may be shared between these types of tissue. Identification of their unique metabolic features is important for a better understanding of biochemical reprogramming in the thyroid nodules. In the majority of works, metabolic profiles of the nodules are compared to the spectra of the macroscopically normal thyroid tissue resected from thyroid cancer patients^9,10,14^. However, little attention has been paid to the metabolic heterogeneity of this control tissue. Recent evidence indicates that the molecular profiles of such tissue are influenced by the factors secreted by the tumor^17,18^. The inflammation related pathways and the diminished expression of the normal development pathways (such as myogenesis and adipogenesis) were identified in the tissues excised from the tumor surroundings in 8 cancer types, including thyroid cancer^17^. Furthermore, the DNA methylation patterns in some normal tissues adjacent to the thyroid lesions were found to be similar to the patterns typical to these lesions^18^. Interestingly, the cancer induced alterations are seen at a substantial distance (few cm) from the tumor margins and even in the contralateral lobe/side^17^.

The role of inflammation in thyroid cancer pathogenesis and prognosis is a matter of an ongoing debate^19,20^. Infiltration of the tumor by inflammatory cells may be related to the background autoimmune diseases (chronic lymphocytic thyroiditis) or may suggest the response of the host to the tumor^21^. Presence of inflammatory cells in the non-malignant tissue from the tumor bearing thyroid gland is often indicated in the pathology reports.

In this study ^1^H HR MAS NMR (proton High-Resolution Magic Angle Spinning Nuclear Magnetic Resonance) spectroscopy was exploited to investigate the distinct and common metabolic features of the malignant and benign thyroid lesions in reference to the non-transformed tissue from the contralateral gland. We hypothesized that the presence of inflammatory cell infiltrate within the control tissue could be reflected in ^1^H HR MAS NMR spectra and should be taken into account in the analysis of metabolic reprogramming in the thyroid lesions.

## Materials and methods

### Patients

The study was approved by the Bioethics Committee at Maria Skłodowska‐Curie National Research Institute of Oncology Gliwice Branch (approval ID 12/2015/1/2016) and informed consent was obtained from all patients. All research was performed in accordance with relevant guidelines and regulations.

The studied group consisted of 141 patients (113 females and 28 males) undergoing surgical thyroidectomy in Maria Skłodowska-Curie National Research Institute of Oncology, Gliwice Branch. Mean age was 53.3 ± 15.8 years. The flow chart of the studied group is presented in Supplementary Fig. S1. 136 tissue samples were obtained from the thyroid lesions and 112 samples from the non-tumoral thyroid tissue in the contralateral lobe. Mean sample weight was 10.0 ± 3.6 mg. The tissue samples were removed during surgical resection. Immediately after collection, the samples were snap-frozen on dry ice and stored at − 80 °C until the HR MAS ^1^H NMR measurements. The final diagnosis was obtained from a routine postoperative histopathological examination of the corresponding surgical specimens. Among 136 samples obtained from the lesions 32 were benign (12 samples—follicular adenoma, 3 samples—Hürtle cell adenoma, 15 samples—nodular goiter, 2 samples—colloid nodule) and 112 were malignant (89 samples—papillary cancer, 5 samples—follicular cancer, 8 samples—medullary cancer, 2 samples—undifferentiated or poorly undifferentiated cancer). In the subsequent analyses only the specimens from the papillary cancer were included in the group of the cancerous samples. The non-tumoral specimens were obtained contra-laterally to 26 benign lesions and 86 malignant ones.

The non-destructive nature of HR MAS NMR spectroscopy allows histopathological evaluation of the samples examined previously with this method. In this study such evaluation was performed to confirm the presence of the cancer cells in the samples collected from the papillary cancer and to gain insight into the cellular composition of the non-tumoral tissue. Although freezing, thawing and magic angle spinning caused the artefactual changes (Supplementary Fig. S2), the post-NMR analysis was performed in 71 of 89 papillary cancer samples and in all samples from the non-transformed tissue. The presence of the cancer cells was confirmed in 38 samples of the papillary cancer. 112 specimens collected from the non-tumoral tissue were classified either as colloid goiter (80 samples) or chronic thyroiditis (32 samples). Finally, 182 samples (38 from the papillary cancer, 32 from the benign lesions and 112 samples from the non-tumoral tissue) were included in the statistical analysis.

### HR MAS NMR

The HR MAS ^1^H NMR experiments were performed on a Bruker Avance III 400 MHz spectrometer (Bruker BioSpin GmbH, Karlsruhe, Germany) operating at a proton frequency of 400.17 MHz equipped with a ^1^H optimized 4-mm ^1^H/^13^C MAS probe. The frozen tissue samples were weighted and placed in 30 μl disposable Kel-F inserts filled with a 5 μl cold solution of 25 mM sodium formate in D_2_0 for the shimming and locking purposes. The inserts were introduced into 4-mm zirconium HR-MAS rotors. Water presaturated 1D proton Carr-Purcell-Meiboom-Gill (CPMG sequence: cpmgpr1d, Bruker) spectra were acquired at 4 °C with a magic angle spinning at 4000 Hz using the following parameters: a relaxation delay of 4 s, an effective echo time of 60 ms, a spectral width of 20 ppm, 65,536 points, an acquisition time of 4.08 s and 256 scans. Assignments of the metabolite signals were performed using literature data^9–15^ and 2D NMR spectra (J-resolved, ^1^H–^1^H TOCSY and ^1^H–^13^C HSQC) acquired for selected samples.

### Data analysis

The raw HR MAS NMR CPMG spectra were apodized with an exponential function (0.7 Hz), Fourier-transformed, phased and baseline corrected using TopSpin 3.1 software (Bruker BioSpin GmbH). The signals were referenced to the formate peak at 8.44 ppm using SpecAlign software (ver. 2.4.1, University of Oxford). The spectra were scaled using a probabilistic quotient normalization technique in the range from 0.8 to 4.7 ppm and logarithmically transformed. The pre-processed data were imported to SIMCA-P 15.0 software (Umetrics; Sweden) for a multivariate analysis. The mean centered and Pareto scaled spectra were subjected to principal component analysis (PCA) and orthogonal partial least squares discriminant analysis (OPLS-DA). The results were interpreted by means of the scores and loadings plots. The combination of the VIP (Variable Importance at Projection) scores and the loadings scaled as the correlation coefficients (pcorr values) was used for identification of the potential biomarkers, as suggested by Wheelock et al.^22^ and recently used in the metabolomic investigation of the thyroid lesions^13^.

The VIP values > 0.7 and |p(corr)| > 0.45 were considered as the inclusion criteria for putative biomarkers. Every latent variable was described by a fraction of the explained variation R2. The fraction of the predicted variation Q2 was determined using seven-fold cross-validation for the supervised OPLS-DA models. The statistical significance of the models was also evaluated using permutation testing (the number of permutations was equal 100) and the cross validation analysis of variance (CV-Anova) at alpha = 0.05.

**Figure 1:**
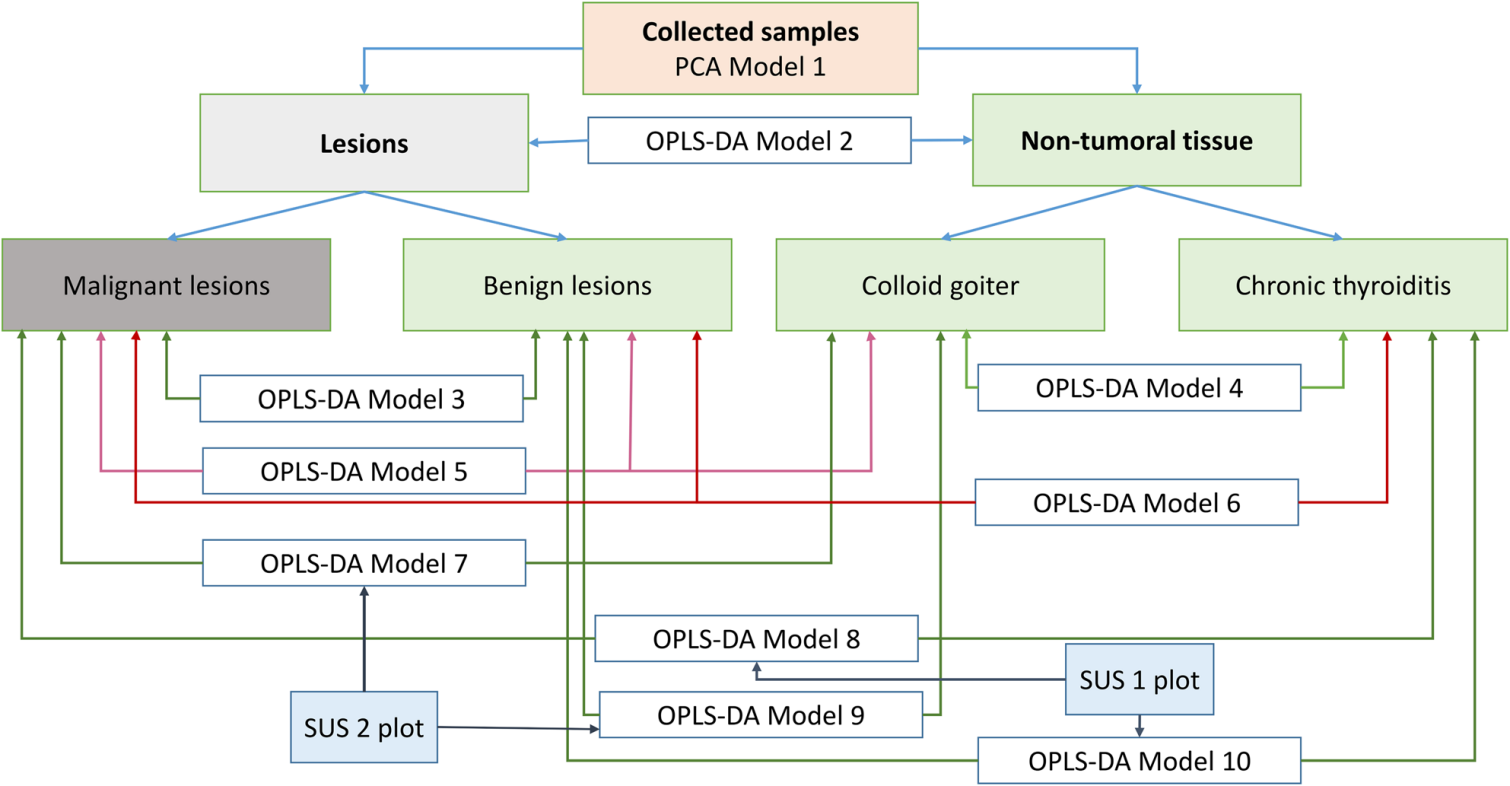
The scheme presenting the constructed multivariate models.

The multivariate analyses were performed according to the scheme presented in Fig. 1.

As a first step, a global PCA analysis of the whole dataset was conducted to inspect the natural clustering of the spectra (Fig. 1, model 1). Then, a supervised OPLS-DA modeling was done to differentiate the spectra acquired from the lesions and from the non-tumoral tissue (model 2). The same technique was also used to separate the benign lesions from the malignant ones (model 3) and to investigate a metabolic heterogeneity of the non-transformed tissue (colloid goiter vs. chronic thyroiditis) (model 4). The three-class OPLS-DA models were built to check the metabolic differences between the malignant lesions, benign lesions and goiter (model 5) and between the malignant lesions, benign lesions and thyroiditis (model 6). However, contrary to the two-class case where the OPLS-DA models always represent the class discrimination along the predictive component, in case of multi-classes the separation may not be along a single axis, thus turning interpretation less straightforward^23^. For this reason, also the two-class models differentiating the malignant lesions from colloid goiter (model 7), the malignant lesions from chronic thyroiditis (model 8), the benign lesions from colloid goiter (model 9) and the benign lesions from chronic thyroiditis (model 10) were constructed (according to Fig. 1). Then, their loadings were compared against each other (7 with 9 and 8 with 10) using the shared and unique structures (SUS) plots to decipher the differences and similarities between these models. The SUS plot analyses were carried using the appropriate modules implemented in SIMCA 15 software (Umetrics; Sweden) and are presented in “Results”.

The SUS plots were constructed by combining the pcorr values obtained for the predictive component [pcorr(1)] from the two two-class models having the same reference group. For the identical profiles, the SUS-plot should have all the points on the diagonal line from the lower left corner to the upper right corner, with R2 = 1.0. Thus, the variables located along the positive “/” diagonal of the plot show the same behavior in both models, those aligned along the negative “\” diagonal display the opposite behavior in the compared models, and, finally, the alignment along the horizontal or vertical axes indicates an effect in one of the models^23^.

Thus, to facilitate the evaluation of the results the SUS plot area was divided into three sections defined by the pcorr(1) values:

- Section 1: the |pcorr(1)| values > 0.45 in both models; the shared metabolic effects.
- Section 2: the |pcorr(1)| values > 0.45 in one model and the |pcorr(1)| values ranging from 0 to 0.3 in the other model; a strong differentiating effect of the metabolites, and
- Section 3: the |pcorr(1)| values > 0.45 in one model and the |pcorr(1)| values ranging from 0.3 to 0.45 in the other model; a medium differentiating effect of the metabolites. The metabolites located in this section were considered typical to one of the evaluated models if univariate analysis of the signal integrals confirmed the specificity.

Kruskal–Wallis test was used in the univariate analysis of the differences in the signal integrals between four groups (malignant lesions, benign lesions, colloid goiter and chronic thyroiditis). When this test showed significant results (p < 0.05), post-host analysis was performed by means of multiple comparisons of mean ranks. The analysis was conducted in Statistica 12.5 software (StatSoft, Tulsa, USA).

The signal integrals were also subjected to the pathway analysis (including the pathway enrichment and the topology analysis) in MetaboAnalyst 4.0 software^24^. The analysis was based on Small Molecule Pathway Database (SMPDB). The pathways were considered important when the pathway impacts were higher than the threshold set to be 0.1 and if the FDR (False Discovery Rate) p values obtained from the enrichment analysis were below 0.05.

## Results

**Figure 2:**
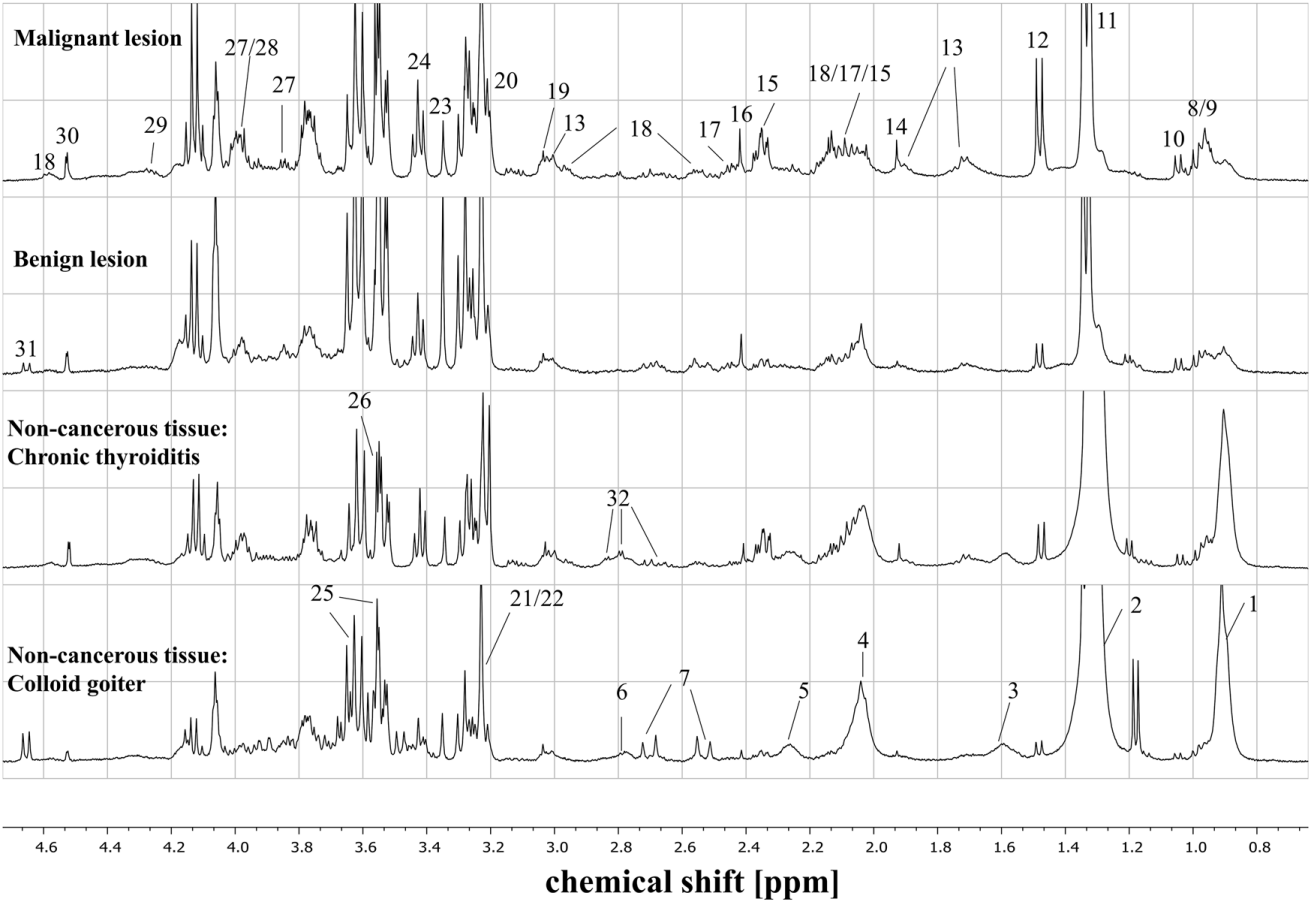
Representative HR MAS ^1^H NMR CPMG spectra of the malignant lesions, benign lesions, chronic thyroiditis (non-tumoral tissue) and colloid goiter (non-tumoral tissue). The assigned signals: 1—fatty acids (FA) 0.9 ppm, 2—fatty acids (FA) 1.3 ppm, 3—fatty acids (FA) 1.59 ppm, 4—fatty acids (FA) 2.03 ppm, 5—fatty acids (FA) 2.25 ppm, 6—fatty acids (FA) 2.77 ppm, 7—citrate (Cit), 8—isoleucine (Ile), 9—leucine (Leu), 10—valine (Val), 11—lactate (Lac), 12—alanine (Ala), 13—lysine (Lys), 14—acetate (Ace), 15—glutamate (Glu), 16—succinate (Suc), 17—glutamine (Gln), 18—glutathione (GSH), 19—creatine (Cre), 20—choline (Cho), 21—phosphocholine (Pho), 22—glycerophosphocholine (GPC), 23—scyllo-inositol (SI), 24—taurine (Tau), 25—myo-inositol (MI), 26—glycine (Gly), 27—serine (Ser), 28—cysteine (Cys), 29—threonine (Thre), 30—ascorbate (Asc), 31—glucose (Glc), 32—aspartate (Asp). The image was created using Statistica 12.5 software (StatSoft, Tulsa, USA). www.statsoft.pl.

Figure 2 presents the representative HR MAS ^1^H NMR CPMG spectra acquired from the thyroid lesions (malignant and benign) and non-tumoral tissue (colloid goiter and chronic thyroiditis). The metabolite signals were assigned based on the 2D HR MAS NMR spectra measured for the selected samples and on the literature data^9–15^.

The ^1^H NMR CPMG spectra of the thyroid samples from the lesions and the adjacent normal appearing tissue were subjected to a multivariate data analysis involving an unsupervised method (PCA) and a supervised modeling (OPLS-DA).

### PCA model 1

**Figure 3:**
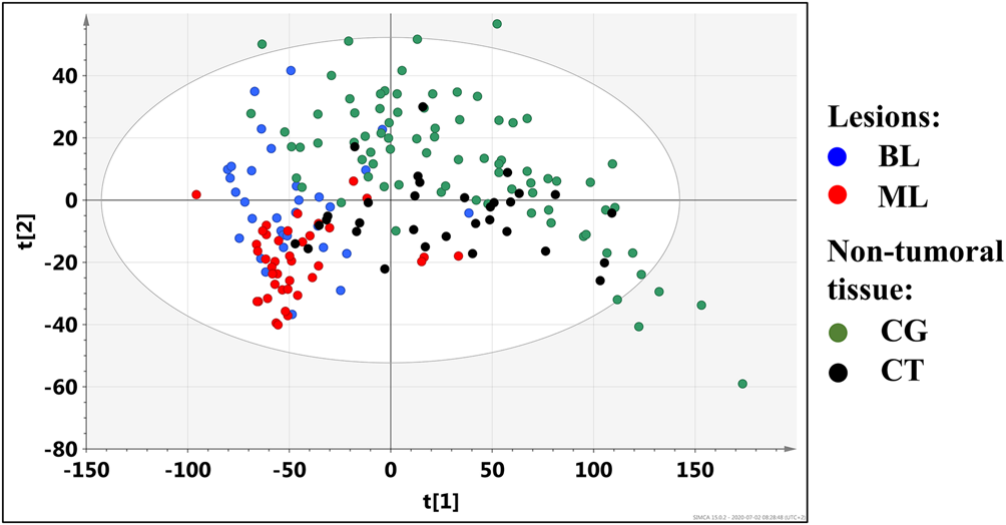
The scores plot obtained from the PCA analysis of the whole dataset (model 1): blue filled circle the benign lesions (BL), red filled circle the malignant lesions (ML), green filled circle colloid goiter (CG, the non-tumoral tissue) and black filled circle chronic thyroiditis (CT, the non-tumoral tissue). The image was created using SIMCA-P 15.0 software package (Umetrics AB, Umeå, Sweden). https://www.sartorius.com.

The scores plot obtained from the PCA analysis of the whole dataset (model 1) is presented in Fig. 3. The first and the second principal components represent 70.3% of the overall variation. The separation between the lesions and the adjacent normal appearing thyroid tissue is clearly seen. Moreover, both groups reveal some heterogeneity. It is expected, as the lesions involve the malignant and benign lesions, whereas the normal appearing tissue group contains the colloid goiter and chronic thyroiditis samples.

### OPLS-DA model 2

**Figure 4:**
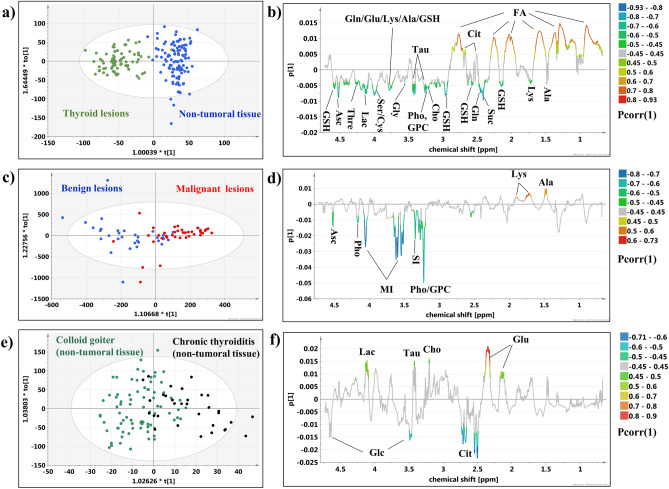
The OPLS-DA models: (**a**) the scores and (**b**) loadings plots obtained from the model 2, (**c**) the scores and (**d**) loadings plots obtained from the model 3, (**e**) the scores and (**f**) loadings plots obtained from the model 4. The signals on the loadings plots are colored according to the pcorr(1) values. The image was created using SIMCA-P 15.0 software package (Umetrics AB, Umeå, Sweden). https://www.sartorius.com.

The metabolic differences between the lesions and the non-tumoral tissue (Fig. 4a) were investigated using the supervised OPLS-DA technique. The analysis resulted in the model 2 build with 1 predictive and 4 orthogonal components (for the predictive component: R2X = 0.33, R2Y = 0.85, Q2 = 0.78, CV-ANOVA p value = 0.0). The separation between the groups (Fig. 4b) is mainly caused by the higher alanine, lysine, glutamine, succinate, glutathione, choline, phosphocholine, taurine, serine/cysteine, glycine, lactate, threonine, ascorbate levels, and the lower lipids’ and citrate signals in the spectra measured from the lesions as compared to the spectra obtained from the normal appearing tissue.

### OPLS-DA model 3

The OPLS-DA technique was also successful in visualization of the metabolic differences between the malignant and benign lesions (Fig. 4c). The model 3 consisted of 1 predictive and 1 orthogonal components (the predictive component: R2X = 0.13, R2Y = 0.50, Q2 = 0.43, CV-ANOVA p value = 1.6e−07) reveals lower phosphocholine/glycerophosphocholine, myo-inositol, scyllo-inositol and ascorbate and higher alanine and lysine in the malignant lesions in comparison to the benign ones (Fig. 4d). The Kruskal–Wallis test followed by the multiple comparisons of the mean ranks confirms these findings with the exception of ascorbate (p = 0.11).

### OPLS-DA model 4

The heterogeneity of the HR MAS NMR spectral profiles within the group of the non-tumoral thyroid tissue samples is seen in the OPLS-DA model 4 differentiating the colloid goiter and chronic thyroiditis (Fig. 4e). The obtained model consisted of 1 predictive and 2 orthogonal components (the predictive component: R2X = 0.05, R2Y = 0.48, Q2 = 0.36, CV-ANOVA p value = 2.0e−08). The loadings plot (Fig. 4f) shows the separation between both groups caused by higher glutamate, choline, taurine and lactate and lower citrate and glucose in thyroiditis than in the goiter samples. The differences in glutamate and citrate were also found to be statistically significant in the univariate analysis.

The cross-validated scores plots obtained from the models 2–4 and the results from a permutation testing are presented in Supplementary Fig. S3.

### OPLS-DA models 5 and 6

The scores plots obtained from the three-class models 5 and 6 show a good separation between the analyzed groups (Supplementary Fig. S4). The analysis produced the following results:

- Model 5 (benign lesions vs. malignant lesions vs. goiter) built from 2 predictive (R2X = 0.41, R2Y = 0.70, Q2 = 0.60, CV-ANOVA p value 1.7e−33) and 2 orthogonal components

and

- Model 6 (benign lesions vs. malignant lesions vs. thyroiditis) containing 2 predictive (R2X = 0.46, R2Y = 0.63, Q2 = 0.54, CV-ANOVA p value 1.1e-18) and 2 orthogonal components.

### OPLS-DA models 7–10

**Figure 5:**
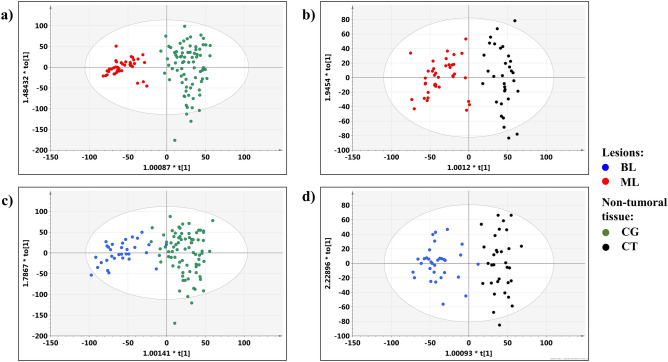
The O-PLS-DA scores plots for the models: (**a**) 7, (**b**) 8, (**c**) 9, (**d**) 10. Red filled circle the malignant lesions (ML), blue filled circle the benign lesions (BL), green filled circle colloid goiter (CG), and black filled circle chronic thyroiditis (CT). The image was created using SIMCA-P 15.0 software package (Umetrics AB, Umeå, Sweden). https://www.sartorius.com.

The scores plots obtained from the models 7–10 are presented in Fig. 5. The OPLS-DA models’ characteristics are as follows:

- Model 7—differentiates the malignant lesions from colloid goiter and is built with 1 predictive (R2X = 0.33, R2Y = 0.88, Q2 = 0.85, extremely small p value obtained from CV-ANOVA rounded to 0.0 by the software) and 2 orthogonal components,
- Model 8—discriminates between chronic thyroiditis and the malignant lesions and is formed with 1 predictive (R2X = 0.42, R2Y = 0.85, Q2 = 0.73, CV-ANOVA p value = 2.0e−16) and 2 orthogonal components,
- Model 9—separates the benign lesions from colloid goiter is consisted from 1 predictive (R2X = 0.31, R2Y = 0.79, Q2 = 0.72, CV-ANOVA p value = 2.5e−26) and 2 orthogonal components,
  and
- Model 10—shows the differences between chronic thyroiditis and the benign lesions; the model structure involves 1 predictive (R2X = 0.37, R2Y = 0.86, Q2 = 0.76, CV-ANOVA p value 1.6e−14) and 3 orthogonal components.

The detailed analysis of the metabolic differences between the models 7 and 9 as well as between the models 8 and 10 was performed using the SUS plots (as indicated in Fig. 1). It displays the shared and unique features between two different classification models that have a common reference.

### SUS plot 1

**Figure 6:**
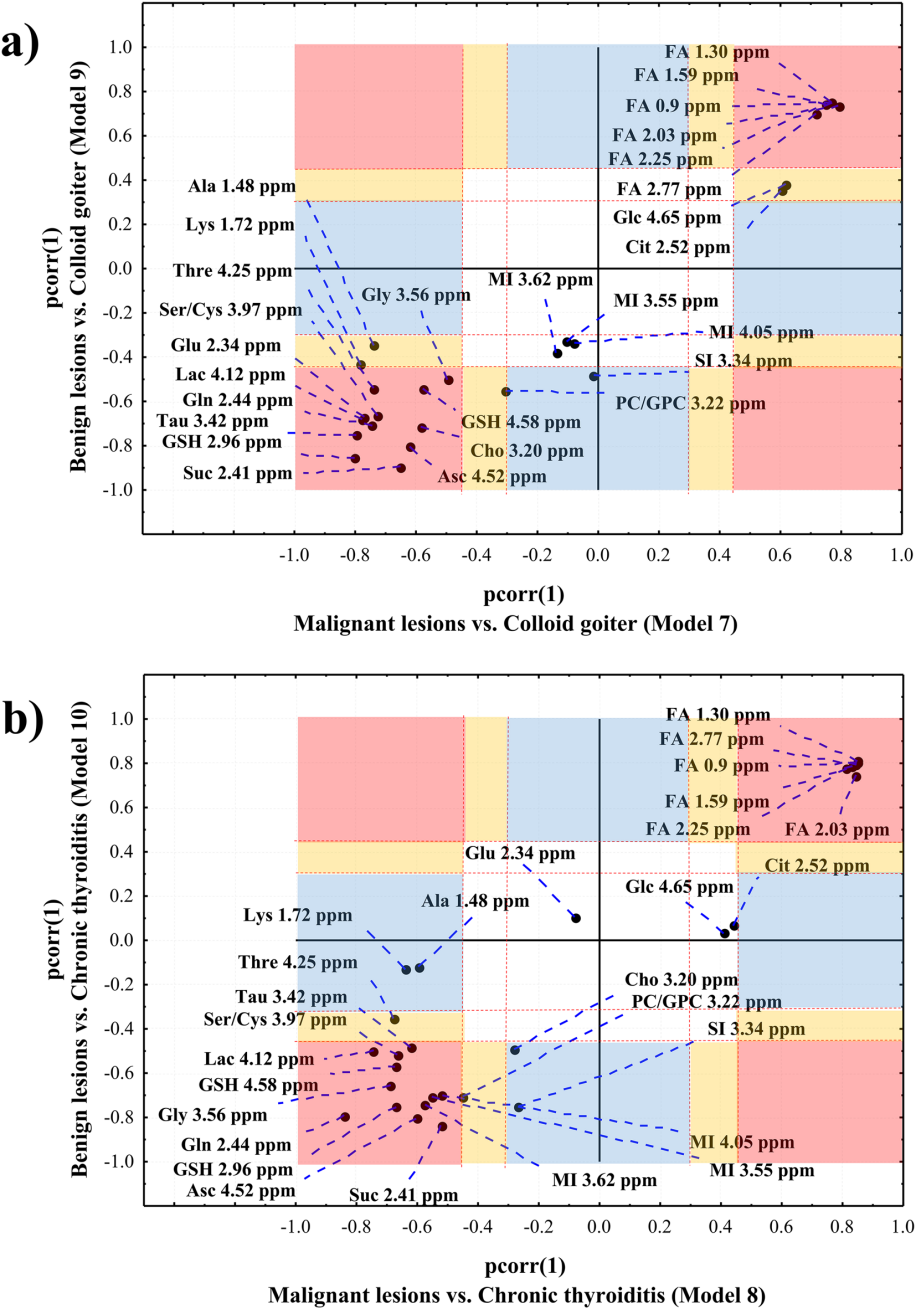
A Shared and Unique Structures (SUS) analysis – a SUS-plot comparing the OPLS-DA models: (**a**) 7 vs 9 and (**b**) 8 vs 10. pink filled square—section 1, |pcorr(1)| > 0.45 in both models, blue filled square—section 2, |pcorr(1)| > 0.45 in one model and 0 < |pcorr(1)| < 0.3 in the other model and yellow filled square—section 3, |pcorr(1)| values > 0.45 in one model and 0.3 < |pcorr(1)| < 0.45 in the other model. Red open square denotes the area where the metabolites do not contribute to the models. The dashed red lines mark the limits of the sections. The metabolites located along the diagonals represent the shared features while the off-diagonal metabolites represent the unique features. The image was created using SIMCA-P 15.0 software package (Umetrics AB, Umeå, Sweden). https://www.sartorius.com.

Figure 6a shows the SUS plot for the OPLS-DA models 7 and 9—the former differentiates the malignant lesions from colloid goiter and the latter separates the benign lesions from colloid goiter. Table 1 gathers the pcorr(1) and VIP values obtained from the models as well as the results of the univariate analysis (the fold-changes and p values). The lower levels of lipids and the higher levels of succinate, glutamine, glutathione, glutamate, choline, taurine, serine/cysteine, glycine, lactate, threonine and ascorbate in the lesions in comparison to the non-malignant tissue are common features of both models. These metabolites are positioned in the section 1 of the SUS plot (Fig. 6a). The higher scyllo-inositol level is typical in the benign lesions in reference to colloid goiter (not observed in the malignant lesions). This metabolite is located in the section 2 of the SUS plot and is characterized by the |pcorr(1)| values > 0.45 in the model 9 and |pcorr(1)| value close to 0 in the model 7. The univariate analysis confirms the specificity of this change. Conversely, alanine and lysine are located in the section 3 and are characterized by |pcorr(1)| > 0.45 in the model 7 and |pcorr(1)| from the range of 0.3–0.45 in the model 9. The univariate analysis reveals the statistical significance of the differences in these metabolites between the malignant lesions and colloid goiter, whereas for the comparison of the benign lesions and the non-cancerous tissue it shows a lack of significance. Therefore, the increased alanine and lysine levels could be considered as the metabolic features typical for the malignant lesions (not observed in the benign lesions) in reference to colloid goiter. The chemical shifts corresponding to citrate, glucose and phosphocholine/glycerophosphocholine also fall in the section 3 of the SUS plot (near a positive diagonal), but the univariate analysis indicates that these metabolites are changed in both lesion types in comparison to colloid goiter. The decrease of citrate and glucose and the increase of phosphocholine/glycerophosphocholine in the lesions in comparison to the non-transformed tissue are the differentiating features shown in Table 1.

**Table 1:** The pcorr (1) and VIP values obtained from the OPLS-DA models 7 and 9, the fold-changes (ratios of median metabolite levels in the lesions to the median metabolite levels in the non-tumoral tissue) and p values from the univariate analysis (post-host analysis by means of multiple comparisons of mean ranks) for the metabolites contributing to the class separation. Positive (negative) pcorr(1) values indicate a relatively higher (lower) metabolite level in the non-tumoral tissue than in the lesions.

Section of the SUS plot 2	Metabolite	Pcorr( 1)		VIP		Fold change p-value	
		Malignant lesions vs colloid goiter (Model 7)	Benign lesions vs colloid goiter (Model 9)	Malignant lesions vs colloid goiter (Model 7)	Benign lesions vs colloid goiter (Model 9)	Malignant lesions/colloid goiter	Benign lesions/colloid goiter
Section 1	FA 0.9 ppm	0.77	0.75	2.06	1.97	0.1 < 0.0000001	0.13 < 0.0000001
	FA 1.30 ppm	0.77	0.75	2.13	2.04	0.08 < 0.0000001	0.12 < 0.0000001
	FA 1.59 ppm	0.75	0.74	1.84	1.83	0.21 < 0.0000001	0.23 < 0.0000001
	FA 2.03 ppm	0.79	0.74	1.70	1.54	0.36 < 0.0000001	0.37 < 0.0000001
	FA 2.77 ppm	0.72	0.70	1.70	1.68	0.37 < 0.0000001	0.37 < 0.0000001
	Glu 2.34 ppm	− 0.77	− 0.67	0.96	0.81	1.45 < 0.0000001	1.20 0.000701
	Suc 2.41 ppm	− 0.65	− 0.89	1.04	1.56	1.5 < 0.0000001	2.06 < 0.0000001
	Gln 2.44 ppm	− 0.74	− 0.71	0.99	0.96	1.59 < 0.0000001	1.55 < 0.0000001
	GSH 2.96 ppm	− 0.80	− 0.85	1.16	1.29	–	–
	GSH 4.58 ppm	− 0.58	− 0.54	0.90	0.88	1.55 < 0.0000001	1.52 0.000005
	Cho 3.20 ppm	− 0.58	− 0.71	1.05	1.27	1.37 0.000006	1.70 < 0.0000001
	Tau 3.42 ppm	− 0.80	− 0.75	1.35	1.29	1.68 < 0.0000001	1.53 0.000002
	Gly 3.56 ppm	− 0.50	− 0.50	0.94	0.97	1.36 0.000152	1.35 0.000555
	Ser/Cys 3.97 ppm	− 0.73	− 0.66	1.20	1.11	1.75 < 0.0000001	1.59 < 0.0000001
	Lac 4.12 ppm	− 0.78	− 0.68	1.34	1.16	1.73 < 0.0000001	1.57 < 0.0000001
	Thre 4.25 ppm	− 0.74	− 0.54	0.86	0.66	1.32 < 0.0000001	1.25 0.000001
	Asc 4.52 ppm	− 0.62	− 0.80	1.07	1.54	1.75 < 0.0000001	2.59 < 0.0000001
Section 2	SI 3.34 ppm	− 0.01	− 0.48	0.70	1.06	0.93 1.0	1.43 0.000150
Section 3	Ala 1.48 ppm	− 0.74	− 0.35	0.94	0.52	1.64* < 0.0000001	1.18* 0.068768
	Lys 1.72 ppm	− 0.78	− 0.43	0.93	0.50	1.31 0.000031	1.04 1.000000
	Cit 2.52 ppm	0.60	0.35	1.06	0.78	0.59 < 0.0000001	0.7 0.003983
	Glc 4.65 ppm	0.62	0.38	1.13	0.80	0.54 0.000001	0.79 0.001107
	PC/GPC 3.22 ppm	− 0.31	− 0.55	0.88	1.18	1.11 0.038559	1.80 < 0.0000001

*Signal intensites were analyzed instead of signal integrals.

### SUS plot 2

To better visualize and interpret the metabolic features from two OPLS-DA models: 8 (chronic thyroiditis vs. the malignant lesions) and 10 (chronic thyroiditis vs. the benign lesions) the SUS plot was employed (Fig. 6b). The pcorr(1) and VIP values obtained from both OPLS-DA models and the results from the univariate analysis (the fold-changes and p-values) for the metabolites contributing to the class separation are listed in Table 2.

**Table 2:** The pcorr(1) and VIP values obtained from the OPLS-DA models 8 and 10, as well as the fold-changes (ratios of median metabolite levels in the lesions to the median metabolite levels in non-tumoral tissue) and p values from the univariate analysis (post-host analysis by means of multiple comparisons of mean ranks) for the metabolites contributing to the class separation. Positive (negative) pcorr(1) values indicate a relatively higher (lower) metabolite level in the non-tumoral tissue than in the lesions.

Section of the SUS plot 1	Metabolite	Pcorr(1)		VIP		Fold change p-value	
		Malignant lesions vs chronic thyroiditis (Model 8)	Benign lesions vs chronic thyroiditis (Model 10)	Malignant lesions vs chronic thyroiditis (Model 8)	Benign lesions vs chronic thyroiditis (Model 10)	Malignant lesions/chronic thyroiditis	Benign lesions/chronic thyroiditis
Section 1	FA 0.9 ppm	0.85	0.80	2.22	2.04	0.08 < 0.0000001	0.1 < 0.0000001
	FA 1.30 ppm	0.85	0.81	2.29	2.10	0.08 < 0.0000001	0.12 < 0.0000001
	FA 1.59 ppm	0.83	0.79	1.95	1.85	0.23 < 0.0000001	0.25 < 0.0000001
	FA 2.03 ppm	0.84	0.75	1.77	1.51	0.36 < 0.0000001	0.37 < 0.0000001
	FA 2.77 ppm	0.85	0.79	1.84	1.75	0.34 < 0.0000001	0.34 < 0.0000001
	Suc 2.41 ppm	− 0.52	− 0.84	0.88	1.45	1.47 0.000014	1.98 < 0.0000001
	Gln 2.44 ppm	− 0.84	− 0.79	1.11	1.07	1.68 < 0.0000001	1.63 < 0.0000001
	GSH 2.96 ppm	− 0.67	− 0.75	0.99	1.12	–	–
	GSH 4.58 ppm	− 0.67	− 0.57	0.91	0.91	1.48 0.000034	1.45 0.002533
	PC/GPC 3.22 ppm	− 0.45	− 0.70	0.76	1.20	1.25 0.010971	2.03 < 0.0000001
	Tau 3.42 ppm	− 0.62	− 0.48	1.02	0.91	1.52 0.000028	1.39 0.043226
	MI 3.55 ppm	− 0.55	− 0.71	0.73	1.10	–	–
	MI 3.62 ppm	− 0.57	− 0.74	0.81	1.19	–	–
	MI 4.05 ppm	− 0.52	− 0.70	0.67	1.07	1.26 0.015601	1.57 < 0.0000001
	Gly 3.56 ppm	− 0.69	− 0.65	0.90	0.89	1.38 0.000039	1.38 0.000111
	Ser/Cys 3.97 ppm	− 0.67	− 0.52	0.98	0.81	1.55 0.000001	1.41 0.004175
	Lac 4.12 ppm	− 0.74	− 0.50	1.12	0.80	1.59 0.000001	1.43 0.000285
	Asc 4.52 ppm	− 0.60	− 0.81	0.96	1.51	1.69 0.000169	2.50 < 0.0000001
Section 2	Ala 1.48 ppm	− 0.60	− 0.12	0.82	0.29	1.46* 0.000238	1.05* 1.0
	Lys 1.72 ppm	− 0.64	− 0.13	0.78	0.30	1.25 0.004356	1 1.000000
	Cho 3.20 ppm	− 0.28	− 0.49	0.69	0.89	1.23 0.069591	1.53 0.000210
	SI 3.34 ppm	− 0.27	− 0.75	0.46	1.28	1.20 0.220164	1.85 < 0.0000001
Section 3	Thre 4.25 ppm	− 0.68	− 0.35	0.80	0.59	1.22 0.000017	1.16 0.023649

*Signal intensites were analyzed instead of signal integrals.

It is evident that the majority of the metabolites exhibit similar behavior in both models. The higher levels of lipids and the lower levels of succinate, glutamine, glutathione, phosphocholine/glycerophospocholine, taurine, myo-inositol, glycine, lactate, serine/cysteine and ascorbate in the non-tumoral tissue in comparison to the lesions were found to fulfill the criteria of |pcorr(1)| > 0.45 and VIP > 0.7 in both models. Of note, all metabolites characterized by the |pcorr(1)| values > 0.45 were confirmed to change significantly between the groups when using the univariate statistics (Table 2).

Alanine and lysine are located in the section 2 (Fig. 6a) characterized by |pcorr(1)| > 0.45 in the model 8 and |pcorr(1)| < 0.3 in the model 10. The higher levels of these metabolites are specific for the malignant lesions (not observed in the benign lesions) in reference to the chronic thyroiditis. The univariate analysis also confirms the specificity of these changes to the model 8.

For choline and scyllo-inositol the characteristic |pcorr(1)| values exceed 0.45 in the model 10 and fall within the range from 0.3 to 0 in the model 8 (section 2). The Kruskal–Wallis test followed by the multiple comparisons of the mean ranks indicates that the increase of these metabolites is a unique feature of the benign lesions in reference to the thyroiditis, not observed in the other model.

Threonine is located in the section 3 of the evaluated SUS plot (Fig. 6b). However, the univariate analysis indicates this metabolite as significantly changed in both malignant and benign lesions in comparison to thyroiditis.

## Analysis of the altered metabolic pathways

The integrals of the signals listed in the Tables 1 and 2 were subjected to the pathway analysis in MetaboAnalyst 4.0 software. Due to the higher contribution of phosphocholine than glycerophosphocholine, the singlet at 3.22 ppm was assumed to represent mainly the former metabolite.

**Figure 7:**
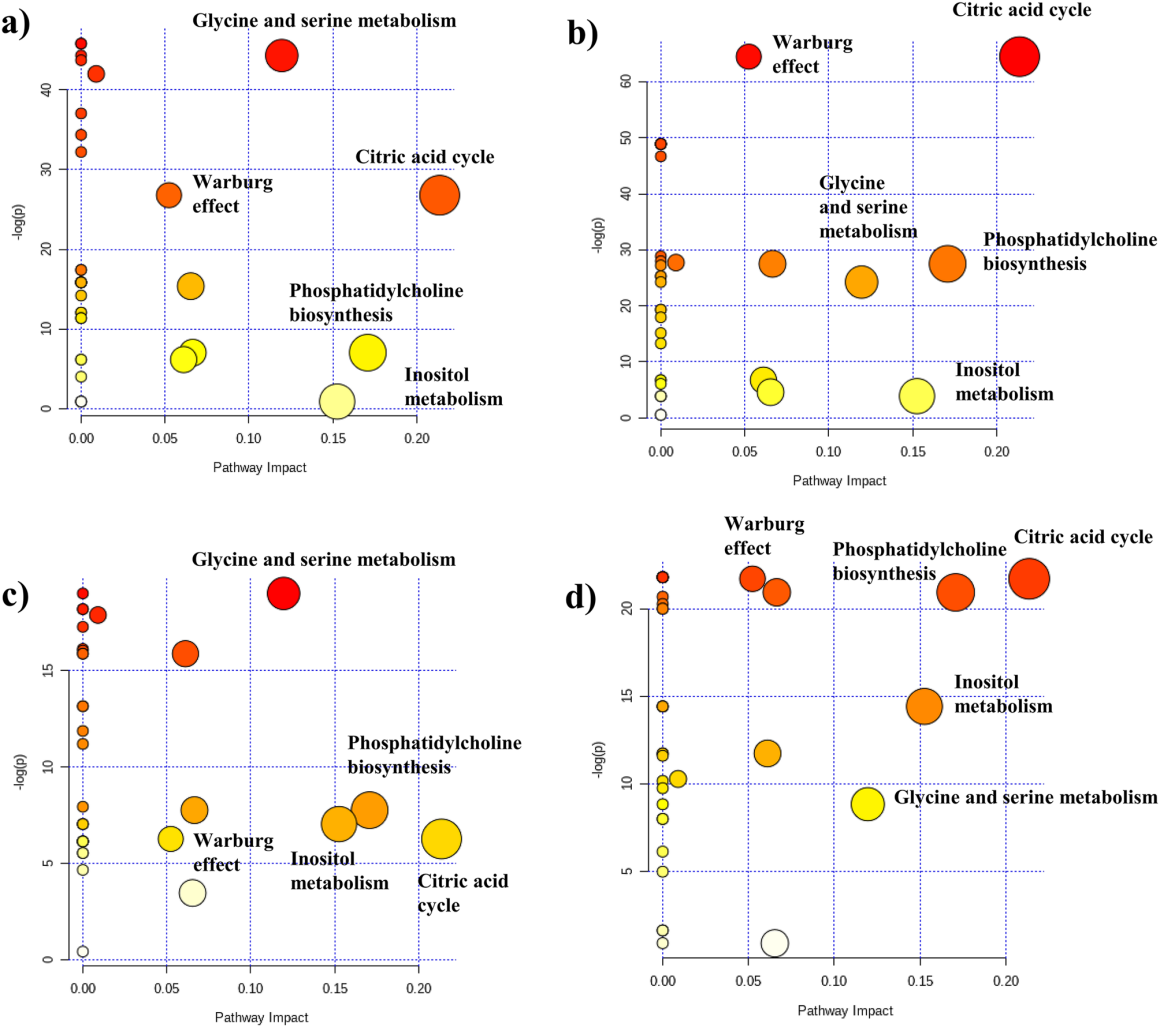
Metabolome views obtained from the pathways analysis presenting the pathways altered in (**a**) the malignant lesions vs. colloid goiter, (**b**) the benign lesions vs. colloid goiter, (**c**) the malignant lesions vs. chronic thyroiditis and (**d**) the benign lesions vs. chronic thyroiditis. On the y-axis, the –log (p) value represents the quantitative perturbation of pathways. On the x-axis, the pathway impact value (the score is from 0 to 1) refers to the centrality of a metabolite in the metabolic network. The node color, varying from yellow to red, is based on its p value, and the node radius is determined on the basis of their pathway impact values. Only pathways with p < 0.05 and/or pathway impact value > 0.10 are considered. The image was created using Metaboanalyst 4.0 software. https://www.metaboanalyst.ca.

The SUS-plots were evaluated in relation to the metabolic pathways that could be disturbed in the compared models. Figure 7a–d shows the metabolome views obtained from the pathway analysis illustrating the metabolic alterations identified in the OPLS-DA models 7–10, while the detailed results are reported in Supplementary Tables S1–S4, ranked according to their probability (with the p values and the impact scores). The metabolic pathway is marked in the figure as significant if a significantly higher number of compounds involved in that pathway is present in the input list than would be expected by a random chance^25,26^.

As seen, 4 pathways—the citric acid cycle, glycine and serine metabolism as well as phosphatidylcholine biosynthesis and inositol metabolism—reveal relatively large impact scores (> 0.1). They can be considered as altered in the thyroid lesions (both malignant and benign) in reference to the non-tumoral tissue (chronic thyroiditis and colloid goiter). However, their corresponding fold enrichment values (Supplementary Table S5) differ markedly for the individual models.

In case of the citric acid cycle the fold enrichment values are higher for the comparisons of the malignant and benign lesions to colloid goiter than to chronic thyroiditis (Supplementary Table S5). This is in accord with the results obtained from the SUS plots: in the SUS plot 1 (where the malignant and benign lesions vs. colloid goiter are shown) citrate falls within the section 3 (Fig. 6a), while in the SUS plot 2 (showing the malignant and benign lesions vs. chronic thyroiditis) citrate is located near the central position of the plot (Fig. 6b), which indicates no meaning of this metabolite in distinguishing the groups involved in the comparison.

The pathway corresponding to the inositol metabolism seems to be perturbed much stronger, both in the benign and malignant lesions in reference to chronic thyroiditis (Fig. 7c,d, Supplementary Table S5) than to colloid goiter (Fig. 7a,b, Supplementary Table S5), where in case of the malignant lesions vs. colloid goiter (Fig. 7a) even the p value limit is not fulfilled (Supplementary Table S1). Similarly, no meaning of the inositol metabolism in the models differentiating malignant and benign lesions vs. colloid goiter is indicated in the SUS plot 1—as myo-inositol falls in the central area of the plot (Fig. 6a). The position of this metabolite in the section 1 of the SUS plot 2 (Fig. 6b, the malignant and benign lesions vs. chronic thyroiditis) points towards an equal role of its metabolism in the malignant and benign lesions referenced to chronic thyroiditis, thus confirming the observation from the pathway enrichment analysis.

The pathway of glycine and serine metabolism is detected as strongly disturbed in all comparisons (Fig. 7a–d). In turn, in both SUS plots these metabolites are located in the section 1 of the strong influence but no differentiating effect (Fig. 6a,b).

The fourth pathway enriched in all comparisons is that of phosphatidylcholine biosynthesis. This process takes place mainly by the Kennedy pathway and the resulting metabolite is used as a material for incorporation into membranes or lipid-derived signaling molecules^27^. Although univariate analysis shows that the signal at 3.22 ppm is higher in both malignant and benign lesions in comparison to colloid goiter and chronic thyroiditis (Tables 1, 2), the fold enrichment values are markedly higher in the comparisons of the benign lesions to the non-tumoral tissue (both colloid goiter and chronic thyroiditis) than in the comparisons of the malignant lesions to this tissue (Supplementary Table S5). It is confirmed by the position of phosphocholine in the SUS plots (Fig. 6). While this metabolite is characterized by |pcorr(1)| values > 0.45 in the models 9 and 10, the lower pcorr(1) values for this metabolite were observed in the models 7 and 8. Choline is another metabolite participating in a phosphatidylcholine pathway. The importance of this metabolite in the comparisons of the malignant and benign lesions to colloid goiter and benign lesions to chronic thyroiditis is visible in the SUS plots (Fig. 6).

## Discussion

The analysis of the HR MAS ^1^H NMR spectra revealed the metabolic diversity between colloid goiter and chronic thyroiditis—two benign conditions frequently encountered in the non-tumoral tissue in patients diagnosed with thyroid lesions. Higher glutamate and lower citrate in the goiter in comparison to thyroiditis were the most apparent differences visible in both multivariate and univariate analysis. Moreover, the multivariate models revealed increased lactate, taurine and choline and decreased glucose in the thyroiditis in comparison to colloid goiter. Of note, the changes in these metabolites were shown to be important in the interpretation of metabolic reprogramming in thyroid cancer^9–15^ and should be taken into account in the construction of the mathematical models enabling differentiation between the tumoral (malignant and benign) and normal appearing tissue.

Increased succinate, glutamine, glutathione, serine/cysteine, ascorbate, lactate, taurine, threonine, glycine, phosphocholine/glycerophosphocholine and decreased lipids were found in both lesion types in comparison to either colloid goiter or chronic thyroiditis. Elevated glutamate and choline, and reduced citrate and glucose were additionally evident in these lesions in reference to goiter, while increased myo-inositol—in comparison to thyroiditis. The malignant lesions were characterized by the higher alanine and lysine levels than colloid goiter and thyroiditis, while scyllo-inositol was uniquely increased in the benign lesions (not in cancer) in comparison to both non-tumoral tissue types. Moreover, the benign lesions presented with the unique increase of choline in reference to thyroiditis (not observed in the cancerous tissue).

One of the well-known metabolic adaptation of cancer to unfavorable conditions in the microenvironment is an increased glucose uptake and conversion of pyruvate (the end product of glycolysis) to lactate via lactate dehydrogenase, even in the presence of adequate oxygen level (Warburg effect)^28^. Another compensatory pathway of pyruvate in tumors is its conversion to alanine via alanine aminotransferase^29^. Decreased glucose, increased lactate and alanine in malignant tumors in comparison to goiter could reflect such processes. The elevated expression of lactate dehydrogenase and glucose transporters in thyroid cancer in reference to the benign lesions and normal tissue was reported^30–32^. Lactate secreted by the stromal compartment is taken up by the malignant cells and converted to pyruvate serving as a substrate for oxidative phosphorylation (the reverse Warburg effect). The papillary cancer cells are claimed to reveal a higher expression of TOMM20 (translocase of outer mitochondrial membrane, a marker of mitochondrial mas) than the non-cancerous tissue, while the stromal tissue in advanced papillary and anaplastic cancer is claimed to overexpress the monocarboxylate transporter 4 (MCT4), a membrane-bound protein being a transporter mediating the outputting lactate from fibroblasts^33^. Interestingly, lactate was not shown to be a differential metabolite between the malignant and benign lesions in our study, in agreement with the findings presented by Rezig et al.^13^.

Although the Warburg effect was initially accepted as a hallmark of cancer, recent data indicate that anaerobic glycolysis may occur in cancer associated fibroblasts rather than in the transformed cells^33^. It is also reported that the metabolism of glycolytic intermediates is involved in tumorigenesis, for example through the glycine and serine metabolic pathways. Serine is an amino acid, required for protein synthesis, fueling glycine biosynthesis and supporting many important metabolic processes^34^. In the cell it is a major substrate for the so-called one-carbon, or folate, cycle which is charged by the conversion of serine in glycine and by the glycine cleavage system. Threonine enters one-carbon metabolism through this glycine cleavage. It gives rise to both glucose and fatty acid precursors and is, thus, characterized as being glycogenic and ketogenic^35^. The relevance of serine/glycine biosynthesis and one-carbon metabolism has recently been claimed to be involved in cancer pathogenesis^36^. The increased serine metabolism might be expected when glycolysis is increased^37,38^, whereas glycine uptake and catabolism is claimed to promote tumorigenesis and malignancy^39^. Of note, the de novo biosynthesis of serine/glycine, is a process that is activated in highly glycolytic cancer cells and the expression rates of the serine/glycine metabolism-related proteins have been found to differ among various thyroid cancers, being higher in papillary thyroid carcinomas and poorly differentiated carcinomas^38^. Recently, the role of glycine (and lactate) as the potential serum marker of papillary thyroid carcinoma has been confirmed in children and adolescents^40^. However, our results indicate that alterations of the metabolism of these amino-acids are not specific to malignant lesions but are also observed in the benign ones. This is in accordance with the results obtained by Xu et al.^16^.

The next pair of amino-acids, alanine and lysine, are also known as the biomarkers of a thyroid malignancy. As reveals from our findings lysine, the solely ketogenic amino-acid, is uniquely increased in the malignant lesions (not in the benign lesions) in comparison to the non-tumoral tissue. We find this amino-acid as also important in discrimination between the malignant and benign lesions. In turn, alanine is a glycogenic amino acid that can serve as an energy source. The appropriate energetic processes involve first the TCA cycle and then gluconeogenesis. The disturbances in energy metabolism are usually observed in thyroid lesions and are described as manifesting in the intensified anaerobic glycolysis and the increased levels of alanine and lactate in the thyroid lesions^14^. Early applications of the liquid state NMR techniques in the studies of intact tissues revealed a significant role of the ratio of the signal intensities at 1.72 ppm (arising from lysine and lipids) and 0.9 ppm (corresponding to the branched chain amino acids and lipids) in the classification of the thyroid samples according to their malignancy^41,42^. However, the reliability of this ratio is reduced by the low resolution of the spectra. The lysine level was shown to be elevated in both malignant and benign lesions in comparison to the adjacent thyroid tissue in the majority of studies applying the HR MAS NMR technique^9,14^. Although we observe the higher levels of lactate and alanine in the malignant lesions in reference to thyroiditis, the glucose concentrations in these groups were similar. Interestingly, energy production in activated inflammatory cells is dependent on the increased glucose utilization (Warburg effect) in addition to oxidative phosphorylation, similarly to the processes observed in tumors^43^. The elevated level of lactate in chronic thyroiditis as compared to colloid goiter revealed by the multivariate analysis is in line with this statement. The results obtained from the enrichment analysis also indicate that the Warburg effect is more relevant alteration for the lesions metabolism in reference to colloid goiter than to chronic thyroiditis (Supplementary Table S5), but its impact—when analyzed alone—is not pronounced (being below the criterion of acceptance) (Fig. 7). Certainly, the situation is much more complex, and many other processes—such as anaerobic glycolysis co-existing with normal TCA function and mitochondrial oxidative phosphorylation pathways—are triggered suggesting a dynamically changing metabolic picture. Enhanced anaerobic glycolysis may be related to the reduced citrate synthesis from pyruvate in mitochondria^36^. This is evident in the tumoral tissue in comparison to colloid goiter, but not to thyroiditis in our work. The discriminative power of citrate between the malignant and benign lesions was not apparent in our data, in contradiction to the findings presented by Deja et al.^10^ and Rezig et al.^13^. However, Torregrossa et al. also did not find this metabolite to be a marker of malignancy^9^.

Glutamine is another compound essential for tumor growth, including thyroid cancer^36,44,45^. This metabolite is involved in many biochemical pathways including glutaminolysis. In this process glutamine is transformed into glutamate via glutaminase—an enzyme overexpressed in thyroid cancer^46^. Glutamate has many fates in tumors, including: a conversion to α-ketoglutarate via glutamate dehydrogenase for the TCA cycle anaplerosis, an involvement in the glutathione synthesis (found to be increased in the lesions compared to the non-tumoral tissue in our study) and the reactions of transamination. A transfer of the amino group from glutamate to pyruvate regulates the alanine synthesis. The increased glutamine and glutamate levels in the thyroid lesions in comparison to the healthy adjacent tissue were often reported in the metabolomics studies^10,14^. Our results show elevated glutamine in the thyroid lesions in reference to colloid goiter and chronic thyroiditis, however, glutamate is increased only in comparison to the former type of the non-tumoral tissue. The concentration of this metabolite in the thyroiditis was similar as in the lesions. The immune cells utilize glutamine at high rates under the metabolic stress conditions^47^. Thus, the increased glutamate level in the thyroiditis as compared to goiter may be related to the enhanced conversion of glutamate from glutamine. However, further studies are required to better understand the biochemical processes underlying these findings.

De novo lipid synthesis is another metabolic adaptation playing an important role in the survival of cancer cells. The increased intensities of the fatty acid signals are frequently observed in HR MAS NMR spectra acquired from various tumors^48,49^. However, recent studies underscore the relevance of fatty acid oxidation in the metabolism of certain cancer types^50^. Lipophylic phenotype of thyroid lesions (both malignant and benign) was observed in the majority of NMR studies^9,11,14,51^. Our results are in agreement with these observations. Although further studies are required to understand the biochemical basis of the decreased lipid signals in the thyroid lesions, a higher metabolic rate and enhanced membrane synthesis could be speculated as possible cause of the observed changes. Interestingly, an overexpression of carnitine palmitoyltransferase 1c (the mitochondrial enzyme controlling the fatty acid oxidation) in papillary thyroid carcinomas compared with the normal thyroid tissues was reported^52^. However, expression of this enzyme in the benign thyroid lesions has not been examined so far.

Activated choline metabolism is a hallmark of carcinogenesis and tumor progression, which leads to the elevated levels of phosphocholine and glycerophosphocholine in almost all types of cancer. Its mechanisms interact with other biochemical pathways such as glycolysis and triglyceride metabolism. High levels of the choline containing compounds are often observed in HR MAS NMR spectra acquired from various tumors reflecting enhanced membrane biosynthesis/degradation and cellular proliferation. However, the results from the published thyroid cancer studies are contradictory rather than conclusive. Tian et al.^14^ report that compared with the healthy adjacent thyroid tissue, the thyroid lesions have significantly higher levels of choline, phosphocholine and glycerophosphocholine (as well as phosphoethanolamine, lactate, GSH, taurine, myo-inositol, inosine, fumarate and amino acids and lower levels of lipids), while other studies indicate the lower levels of choline and phosphocholine (as well as myo-inositol and scyllo-inositol) in the malignant tumors than in the adjacent thyroid tissue^9,11^. In our study univariate analysis revealed the signal at 3.22 ppm to be increased both in the malignant and benign lesions in comparison to the non-transformed tissue. However, the location of this metabolite in the SUS plots (Fig. 6) and the results from enrichment analysis (Supplementary Table S5) indicate that the alterations of this metabolite are more relevant to the benign lesions than to the malignant ones. Of note, the application of ^31^P HR MAS NMR in the studies of thyroid lesions would provide a better insight into the alterations of the Kennedy pathway due to a lower spectral overlap of various choline containing compounds (phosphocholine vs. glycerophosphocholine).

A typical metabolic feature of the benign lesions (not observed in cancer) referenced to both goiter and thyroiditis is the scyllo-inositol increase. Our finding is confirmed by other authors^9,13^ reporting higher concentration of this metabolite in the benign tumors than in the malignant ones, paralleled with the myo-inositol increase—such metabolic changes indicate the disturbances in tumor osmoregulation. In our study myo-inositol plays an important role in both malignant and benign tumors when referenced to thyroiditis but not to goiter. This compound is a precursor in the synthesis of phosphoinositides and contributes in the TSH signaling and to autoimmune disorders (such as chronic thyroiditis)^53^. Furthermore, the members of phosphoinositide-3 kinase group of enzymes exert a regulative effect on lymphocyte metabolism, which in turn influences the myo-inositol levels^53^. However, further studies are required to explain these complex metabolic processes leading to the differences in the HR MAS NMR-visible myo-inositol signals between the thyroid tumors and thyroiditis.

The multivariate models constructed in this study revealed a relevant role of ascorbate in differentiation between the malignant and benign lesions. The reduced level of the vitamin C was associated with malignancy. Similar result was also obtained in the study classifying the indeterminate follicular lesions^13^. Interestingly, an inverse correlation between the ascorbate content and the HIF-1 pathway score in thyroid cancer cells was demonstrated by Jóźwiak et al.^54^.

Our study shows the importance of post-NMR histopathological evaluation of cancer and normal tissue samples in the interpretation of metabolic reprograming in thyroid lesions. However, greater efforts should be undertaken to better preserve the integrity of the samples by using slow spinning HR MAS NMR techniques. Renault et al. found that minimization of the volume of the chamber and its positioning at the coil centre with an insert placed at the top of the rotor enables to obtain good quality spectra at spinning frequencies of 500 Hz^55^. Another important issue for future investigation could be the comparison of HR MAS NMR metabolic profiles of the thyroid lesions to the matched reference spectra of the normal tissue excised both from the contra-lateral and ipsilateral thyroid lobes. This could provide a direct insight into the tumour induced metabolic alterations in the non-malignant tissue.

In conclusion, the metabolic features shared between the benign and malignant thyroid lesions in reference to the contralateral non-tumoral tissue (both colloid goiter and chronic thyroiditis) include: increased succinate, glutamine, glutathione, serine/cysteine, ascorbate, lactate, taurine, threonine, glycine, phosphocholine/glycerophosphocholine and decreased lipids. Elevated glutamate and choline, and reduced citrate and glucose were additionally evident in these lesions in reference to goiter, while increased myo-inositol—in comparison to thyroiditis. The unique features of malignant lesions in comparison to the non-transformed tissue (both colloid goiter and chronic thyroiditis) include the higher alanine and lysine levels, while scyllo-inositol was found to be distinctly increased in the benign lesions (not in cancer) in comparison to both non-tumoral tissue types. Moreover, the benign lesions presented with the unique increase of choline in reference to thyroiditis (not observed in the cancerous tissue).

Metabolic heterogeneity of the non-tumoral tissue with respect to the presence of the inflammatory cell infiltrate should be considered in the analysis of metabolic reprogramming in thyroid lesions.

## Supplementary Information

Supplementary Information

## Data Availability

The datasets generated during and/or analysed during the current study are available from the corresponding author on reasonable request.
