# Supplementary material for: Shared and unique metabolic features of the malignant and benign thyroid lesions determined with use of 1H HR MAS NMR spectroscopy
Source: Sci Rep. 2021 Jan 14;11:1344. doi: 10.1038/s41598-020-79565-8 (PMC7809111; doi:10.1038/s41598-020-79565-8)
Supplement: Supplementary file 1 — Supplementary Information [file 41598_2020_79565_MOESM1_ESM.pdf]

Supplementary information

Shared and unique metabolic features of the malignant and benign thyroid lesions determined with use of ^{1}H HR MAS NMR spectroscopy

Agnieszka Skorupa[1*], Mateusz Ciszek[1], Ewa Chmielik[2], Łukasz Boguszewicz[1], Małgorzata Oczko – Wojciechowska[3], Małgorzata Kowalska[3], Dagmara Rusinek[3], Tomasz Tyszkiewicz[3], Aneta Kluczewska – Gałka[4], Agnieszka Czarniecka[5], Barbara Jarząb[4], Maria Sokół[1]

[1]Department of Medical Physics, Maria Skłodowska - Curie National Research Institute of Oncology, Gliwice Branch, 44-101 Gliwice, Poland;

[2]Tumor Pathology Department, Maria Skłodowska - Curie National Research Institute of Oncology, Gliwice Branch, 44-101 Gliwice, Poland;

[3]Department of Genetic and Molecular Diagnostics of Cancer, Maria Skłodowska - Curie National Research Institute of Oncology, Gliwice Branch, 44-101 Gliwice, Poland

[4]Department of Nuclear Medicine and Endocrine Oncology, Maria Skłodowska - Curie National Research Institute of Oncology, Gliwice Branch, 44-101 Gliwice, Poland

[5]The Oncologic and Reconstructive Surgery Clinic, Maria Skłodowska - Curie National Research Institute of Oncology, Gliwice Branch, 44-101 Gliwice, Poland;

*Corresponding author: agnieszka.skorupa@io.gliwice.pl

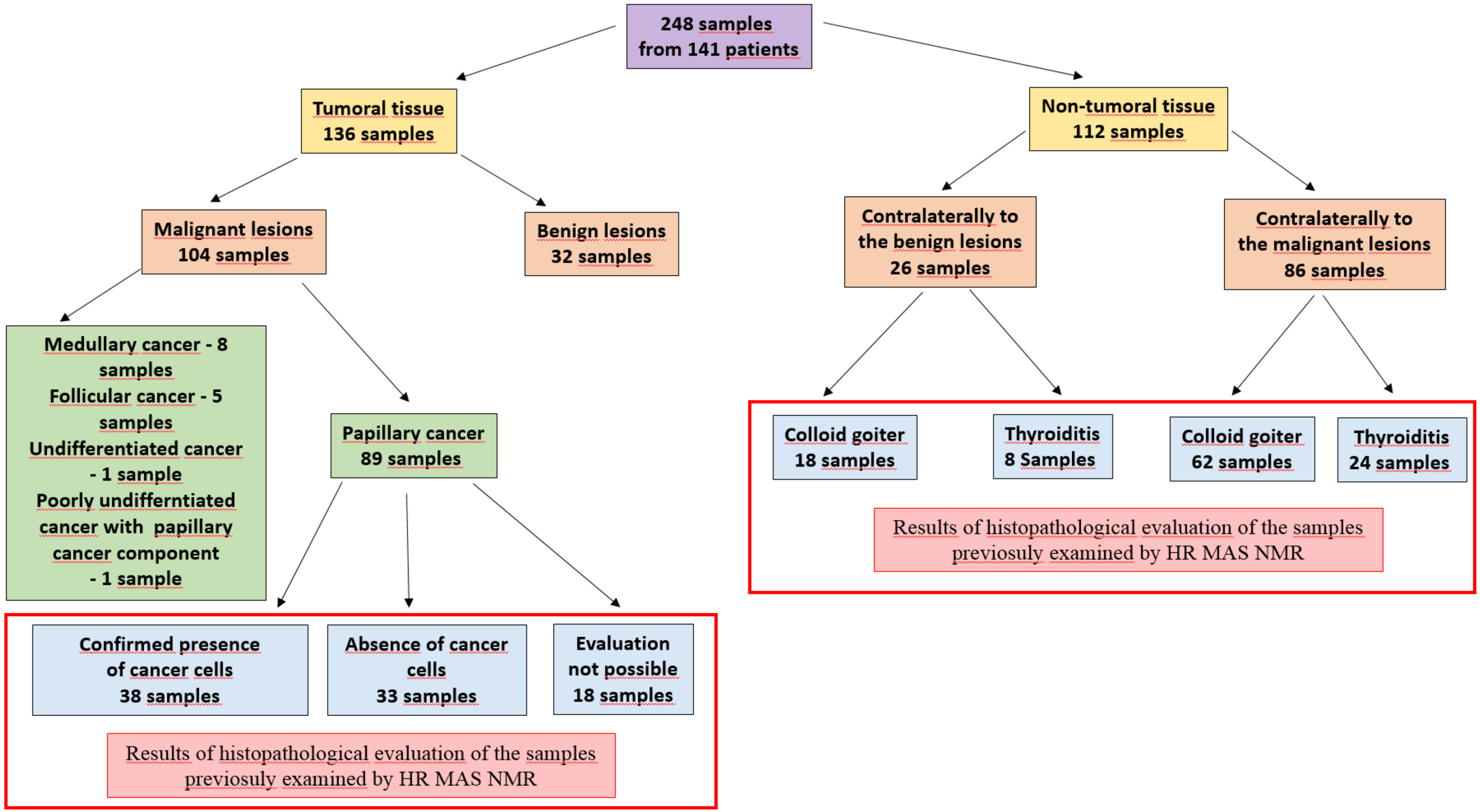

Supplementary Figure S1. The flow chart of the studied group.

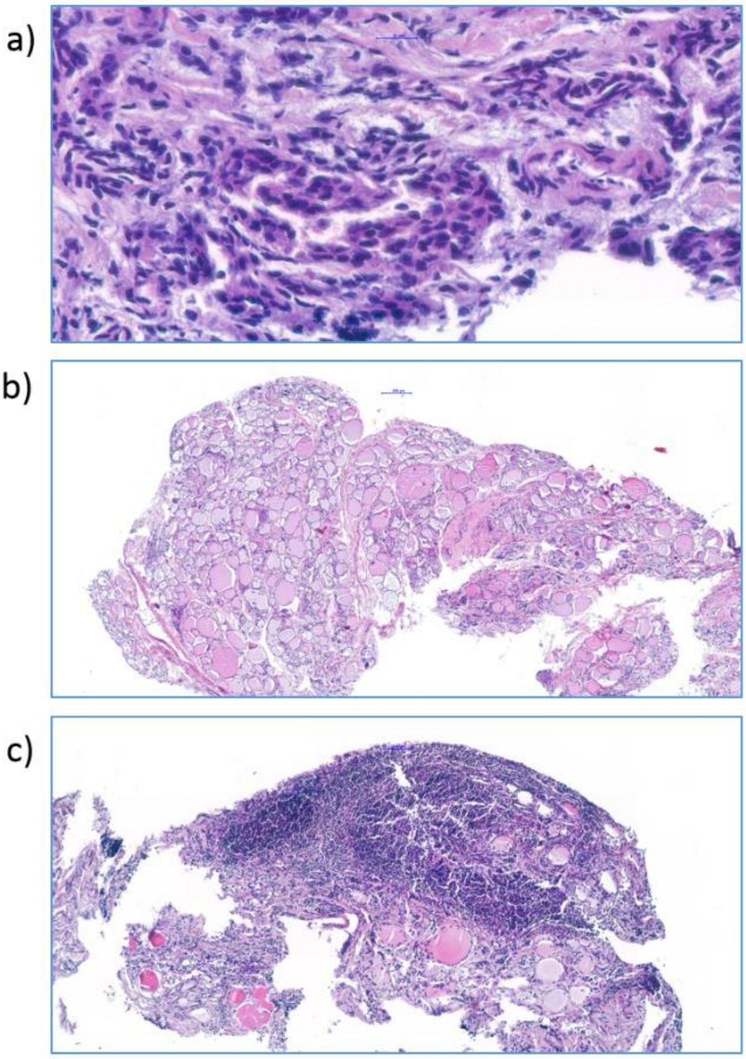

Supplementary Figure S2. H&E staining of samples collected from (a) papillary thyroid cancer, (b) colloid goiter (non-tumoral tissue) and (c) chronic thyroiditis (non-tumoral tissue). In the tissue samples of the papillary carcinomas analyzed histologically after the HR MAS NMR measurements, the architectural arrangements of the cancer cells were observed to be distorted: the cell nuclei become more oval and lined, similarly, the nucleoli are found to change their shape. In case of colloid goiter, the thyrocyte cell nuclei may contract, whereas the colloid may occasionally condense. The least cellular changes were observed in chronic thyroiditis.

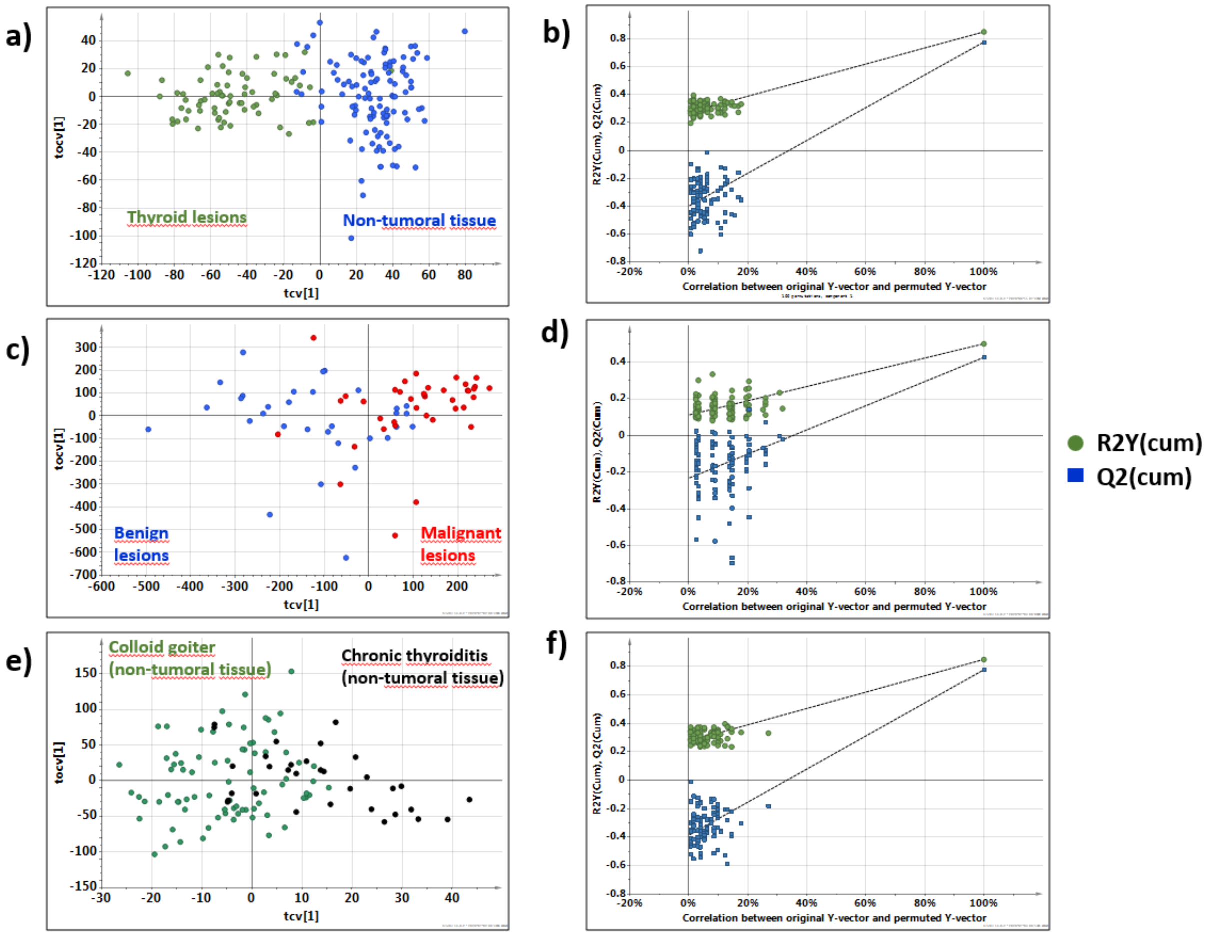

Supplementary Figure S3. The cross-validated scores plots (a) and results from permutation testing (b) obtained for the model 2, the cross-validated scores plots (c) and results from permutation testing (d) obtained for the model 3, the cross-validated scores plots (e) and results from permutation testing (f) obtained for the model 4.

R2Y(cum)- cumulative fraction of the Y variation explained by the model

Q2(cum) - cumulative fraction of the Y variation predicted by the model

The image was created using SIMCA-P 15.0 software package (Umetrics AB, Umeå, Sweden). https://www.sartorius.com

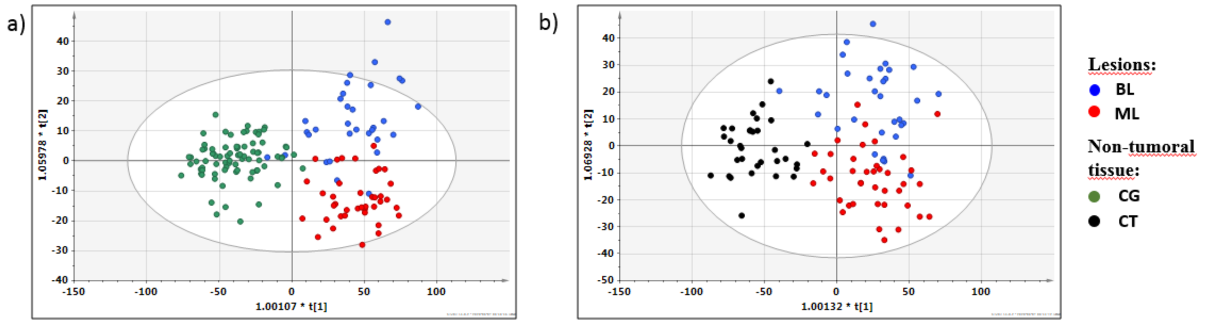

Supplementary Figure S4. The O-PLS-DA scores plots for the models: (a) 5, and (b) 6. ● the malignant lesions (ML), ● the benign lesions (BL), ● colloid goiter (CG), ● chronic thyroiditis (CT).

The image was created using SIMCA-P 15.0 software package (Umetrics AB, Umeå, Sweden). https://www.sartorius.com

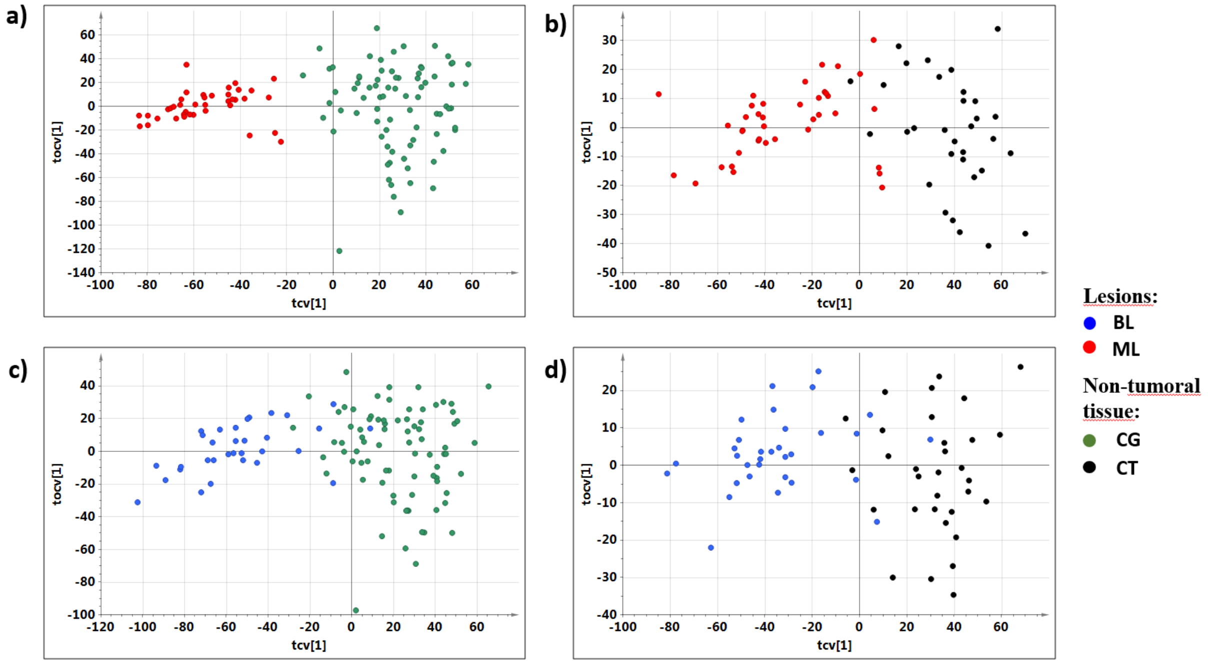

Supplementary Figure S5. The O-PLS-DA cross-validated scores plots for the models: (a) 7, (b) 8, (c) 9, (d) 10. ● the malignant lesions (ML), ● the benign lesions (BL), ● colloid goiter (CG), and ● chronic thyroiditis (CT).

The image was created using SIMCA-P 15.0 software package (Umetrics AB, Umeå, Sweden). https://www.sartorius.com

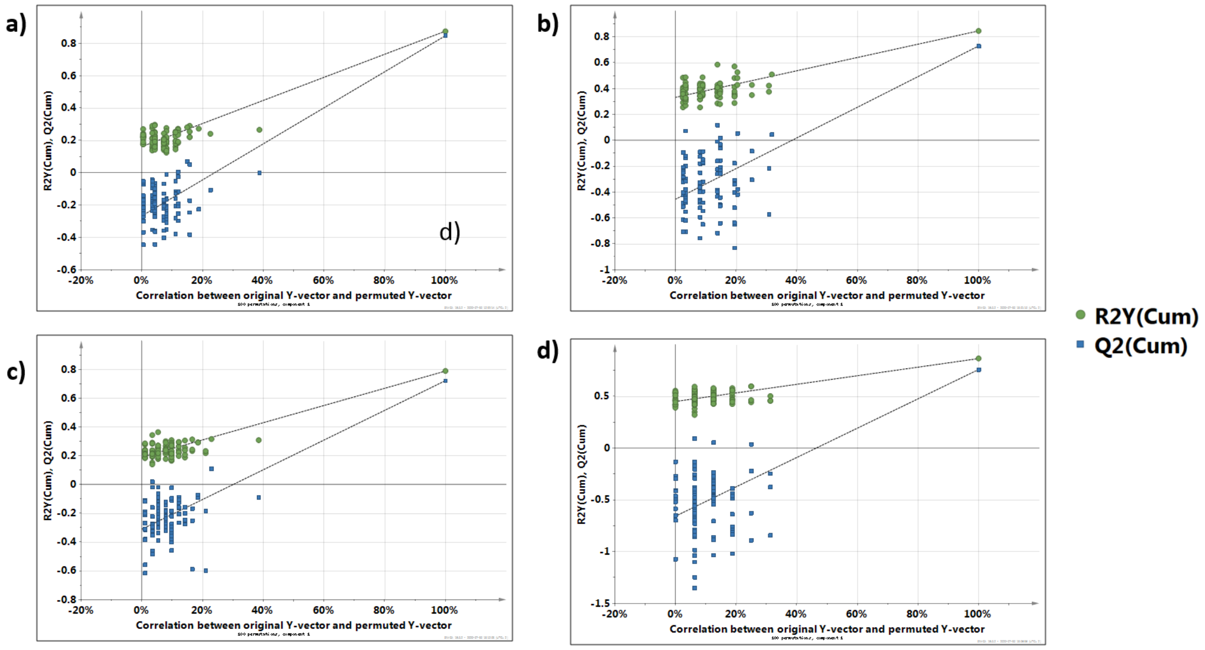

Supplementary Figure S6. Results from a permutation test obtained from O-PLS-DA models: a) 7, (b) 8, (c) 9, (d) 10.

R2Y(cum)- cumulative fraction of the Y variation explained by the model,

Q2(cum) - cumulative fraction of the Y variation predicted by the model.

The image was created using SIMCA-P 15.0 software package (Umetrics AB, Umeå, Sweden). https://www.sartorius.com

	Total Cmpd	Hits	Raw p	-log(p)	Holm adjust	FDR	Impact
Selenoamino Acid Metabolism	28	1	1.24E-20	4.58E+01	4.36E-19	2.18E-19	0.00
Homocysteine Degradation	7	1	1.24E-20	4.58E+01	4.36E-19	2.18E-19	0.00
Glycine and Serine Metabolism	50	2	5.58E-20	4.43E+01	1.84E-18	4.88E-19	0.12
Ammonia Recycling	25	2	5.58E-20	4.43E+01	1.84E-18	4.88E-19	0.00
Phosphatidylethanolamine Biosynthesis	13	2	1.01E-19	4.37E+01	3.13E-18	7.07E-19	0.00
Methionine Metabolism	39	3	5.61E-19	4.20E+01	1.68E-17	3.27E-18	0.01
Threonine and 2-Oxobutanoate Degradation	13	1	8.02E-17	3.71E+01	2.33E-15	4.01E-16	0.00
Cysteine Metabolism	24	1	1.18E-15	3.44E+01	3.30E-14	5.15E-15	0.00
Bile Acid Biosynthesis	59	3	1.02E-14	3.22E+01	2.74E-13	3.95E-14	0.00
Citric Acid Cycle	26	2	2.32E-12	2.68E+01	6.03E-11	7.38E-12	0.21
Warburg Effect	49	2	2.32E-12	2.68E+01	6.03E-11	7.38E-12	0.05
Betaine Metabolism	18	1	2.78E-08	1.74E+01	6.67E-07	7.51E-08	0.00
Carnitine Synthesis	16	3	2.79E-08	1.74E+01	6.67E-07	7.51E-08	0.00
Oxidation of Branched Chain Fatty Acids	22	1	1.33E-07	1.58E+01	2.92E-06	2.44E-07	0.00
Valine, Leucine and Isoleucine Degradation	51	1	1.33E-07	1.58E+01	2.92E-06	2.44E-07	0.00
Ketone Body Metabolism	12	1	1.33E-07	1.58E+01	2.92E-06	2.44E-07	0.00
Butyrate Metabolism	16	1	1.33E-07	1.58E+01	2.92E-06	2.44E-07	0.00
Mitochondrial Electron Transport Chain	15	1	1.33E-07	1.58E+01	2.92E-06	2.44E-07	0.00
Phytanic Acid Peroxisomal Oxidation	19	1	1.33E-07	1.58E+01	2.92E-06	2.44E-07	0.00
Transfer of Acetyl Groups into Mitochondria	18	1	2.13E-07	1.54E+01	3.41E-06	3.74E-07	0.07
Sphingolipid Metabolism	36	2	6.89E-07	1.42E+01	1.03E-05	1.15E-06	0.00
Lysine Degradation	20	1	5.88E-06	1.20E+01	8.23E-05	8.94E-06	0.00
Biotin Metabolism	7	1	5.88E-06	1.20E+01	8.23E-05	8.94E-06	0.00
Arginine and Proline Metabolism	48	2	1.17E-05	1.14E+01	1.41E-04	1.64E-05	0.00
Glutamate Metabolism	45	2	1.17E-05	1.14E+01	1.41E-04	1.64E-05	0.00
Phosphatidylcholine Biosynthesis	18	2	9.03E-04	7.01E+00	9.03E-03	1.17E-03	0.17
Phospholipid Biosynthesis	25	2	9.03E-04	7.01E+00	9.03E-03	1.17E-03	0.07
Glutathione Metabolism	19	1	2.16E-03	6.14E+00	1.73E-02	2.52E-03	0.06
Purine Metabolism	63	1	2.16E-03	6.14E+00	1.73E-02	2.52E-03	0.00
Alanine Metabolism	14	1	2.16E-03	6.14E+00	1.73E-02	2.52E-03	0.00
Porphyrin Metabolism	36	2	1.85E-02	3.99E+00	9.26E-02	2.09E-02	0.00
Inositol Metabolism	28	1	4.20E-01	8.67E-01	1.00E+00	4.20E-01	0.15
Galactose Metabolism	31	1	4.20E-01	8.67E-01	1.00E+00	4.20E-01	0.00
Inositol Phosphate Metabolism	22	1	4.20E-01	8.67E-01	1.00E+00	4.20E-01	0.00
Phosphatidylinositol Phosphate Metabolism	14	1	4.20E-01	8.67E-01	1.00E+00	4.20E-01	0.00

Supplementary Table S1. The perturbed metabolic pathways in the malignant lesions vs. colloid goiter. The table was created using Metaboanalyst 4.0 software. https://www.metaboanalyst.ca.

	Total Cmpd	Hits	Raw p	-log(p)	Holm adjust	FDR	Impact
Citric Acid Cycle	26	2	9.93E-29	6.45E+01	3.48E-27	1.74E-27	0.21
Warburg Effect	49	2	9.93E-29	6.45E+01	3.48E-27	1.74E-27	0.05
Oxidation of Branched Chain Fatty Acids	22	1	5.87E-22	4.89E+01	1.94E-20	2.57E-21	0.00
Valine, Leucine and Isoleucine Degradation	51	1	5.87E-22	4.89E+01	1.94E-20	2.57E-21	0.00
Ketone Body Metabolism	12	1	5.87E-22	4.89E+01	1.94E-20	2.57E-21	0.00
Butyrate Metabolism	16	1	5.87E-22	4.89E+01	1.94E-20	2.57E-21	0.00
Mitochondrial Electron Transport Chain	15	1	5.87E-22	4.89E+01	1.94E-20	2.57E-21	0.00
Phytanic Acid Peroxisomal Oxidation	19	1	5.87E-22	4.89E+01	1.94E-20	2.57E-21	0.00
Carnitine Synthesis	16	3	5.37E-21	4.67E+01	1.45E-19	2.09E-20	0.00
Phosphatidylethanolamine Biosynthesis	13	2	3.00E-13	2.88E+01	7.81E-12	1.05E-12	0.00
Sphingolipid Metabolism	36	2	7.16E-13	2.80E+01	1.79E-11	2.28E-12	0.00
Methionine Metabolism	39	3	9.21E-13	2.77E+01	2.21E-11	2.69E-12	0.01
Phosphatidylcholine Biosynthesis	18	2	1.15E-12	2.75E+01	2.66E-11	2.89E-12	0.17
Phospholipid Biosynthesis	25	2	1.15E-12	2.75E+01	2.66E-11	2.89E-12	0.07
Betaine Metabolism	18	1	1.52E-12	2.72E+01	3.20E-11	3.56E-12	0.00
Selenoamino Acid Metabolism	28	1	1.06E-11	2.53E+01	2.12E-10	2.18E-11	0.00
Homocysteine Degradation	7	1	1.06E-11	2.53E+01	2.12E-10	2.18E-11	0.00
Glycine and Serine Metabolism	50	2	3.00E-11	2.42E+01	5.40E-10	5.52E-11	0.12
Ammonia Recycling	25	2	3.00E-11	2.42E+01	5.40E-10	5.52E-11	0.00
Arginine and Proline Metabolism	48	2	4.15E-09	1.93E+01	6.63E-08	6.91E-09	0.00
Glutamate Metabolism	45	2	4.15E-09	1.93E+01	6.63E-08	6.91E-09	0.00
Threonine and 2-Oxobutanoate Degradation	13	1	1.62E-08	1.79E+01	2.27E-07	2.58E-08	0.00
Bile Acid Biosynthesis	59	3	2.67E-07	1.51E+01	3.47E-06	4.07E-07	0.00
Cysteine Metabolism	24	1	1.72E-06	1.33E+01	2.06E-05	2.50E-06	0.00
Glutathione Metabolism	19	1	1.20E-03	6.72E+00	1.32E-02	1.56E-03	0.06
Purine Metabolism	63	1	1.20E-03	6.72E+00	1.32E-02	1.56E-03	0.00
Alanine Metabolism	14	1	1.20E-03	6.72E+00	1.32E-02	1.56E-03	0.00
Porphyrin Metabolism	36	2	2.31E-03	6.07E+00	1.85E-02	2.89E-03	0.00
Transfer of Acetyl Groups into Mitochondria	18	1	1.03E-02	4.57E+00	7.22E-02	1.24E-02	0.07
Inositol Metabolism	28	1	2.09E-02	3.87E+00	1.26E-01	2.22E-02	0.15
Galactose Metabolism	31	1	2.09E-02	3.87E+00	1.26E-01	2.22E-02	0.00
Inositol Phosphate Metabolism	22	1	2.09E-02	3.87E+00	1.26E-01	2.22E-02	0.00
Phosphatidylinositol Phosphate Metabolism	14	1	2.09E-02	3.87E+00	1.26E-01	2.22E-02	0.00
Lysine Degradation	20	1	5.89E-01	5.29E-01	1.00E+00	5.89E-01	0.00
Biotin Metabolism	7	1	5.89E-01	5.29E-01	1.00E+00	5.89E-01	0.00

Supplementary Table S2. The perturbed metabolic pathways in the benign lesions vs colloid goiter. The table was created using Metaboanalyst 4.0 software. https://www.metaboanalyst.ca.

	Total Cmpd	Hits	Raw p	-log(p)	Holm adjust	FDR	Impact
Glycine and Serine Metabolism	50	2	5.49E-09	1.90E+01	1.92E-07	9.61E-08	0.12
Ammonia Recycling	25	2	5.49E-09	1.90E+01	1.92E-07	9.61E-08	0.00
Selenoamino Acid Metabolism	28	1	1.24E-08	1.82E+01	4.09E-07	1.09E-07	0.00
Homocysteine Degradation	7	1	1.24E-08	1.82E+01	4.09E-07	1.09E-07	0.00
Methionine Metabolism	39	3	1.68E-08	1.79E+01	5.22E-07	1.18E-07	0.01
Phosphatidylethanolamine Biosynthesis	13	2	3.12E-08	1.73E+01	9.36E-07	1.82E-07	0.00
Threonine and 2-Oxobutanoate Degradation	13	1	9.80E-08	1.61E+01	2.84E-06	4.00E-07	0.00
Carnitine Synthesis	16	3	1.07E-07	1.61E+01	2.99E-06	4.00E-07	0.00
Glutathione Metabolism	19	1	1.26E-07	1.59E+01	3.39E-06	4.00E-07	0.06
Purine Metabolism	63	1	1.26E-07	1.59E+01	3.39E-06	4.00E-07	0.00
Alanine Metabolism	14	1	1.26E-07	1.59E+01	3.39E-06	4.00E-07	0.00
Arginine and Proline Metabolism	48	2	1.92E-06	1.32E+01	4.61E-05	5.18E-06	0.00
Glutamate Metabolism	45	2	1.92E-06	1.32E+01	4.61E-05	5.18E-06	0.00
Bile Acid Biosynthesis	59	3	6.95E-06	1.19E+01	1.53E-04	1.74E-05	0.00
Sphingolipid Metabolism	36	2	1.37E-05	1.12E+01	2.87E-04	3.19E-05	0.00
Porphyrin Metabolism	36	2	3.57E-04	7.94E+00	7.14E-03	7.80E-04	0.00
Phosphatidylcholine Biosynthesis	18	2	4.26E-04	7.76E+00	8.09E-03	8.28E-04	0.17
Phospholipid Biosynthesis	25	2	4.26E-04	7.76E+00	8.09E-03	8.28E-04	0.07
Inositol Metabolism	28	1	8.78E-04	7.04E+00	1.49E-02	1.40E-03	0.15
Galactose Metabolism	31	1	8.78E-04	7.04E+00	1.49E-02	1.40E-03	0.00
Inositol Phosphate Metabolism	22	1	8.78E-04	7.04E+00	1.49E-02	1.40E-03	0.00
Phosphatidylinositol Phosphate Metabolism	14	1	8.78E-04	7.04E+00	1.49E-02	1.40E-03	0.00
Citric Acid Cycle	26	2	1.90E-03	6.27E+00	2.47E-02	2.52E-03	0.21
Warburg Effect	49	2	1.90E-03	6.27E+00	2.47E-02	2.52E-03	0.05
Oxidation of Branched Chain Fatty Acids	22	1	2.16E-03	6.14E+00	2.47E-02	2.52E-03	0.00
Valine, Leucine and Isoleucine Degradation	51	1	2.16E-03	6.14E+00	2.47E-02	2.52E-03	0.00
Ketone Body Metabolism	12	1	2.16E-03	6.14E+00	2.47E-02	2.52E-03	0.00
Butyrate Metabolism	16	1	2.16E-03	6.14E+00	2.47E-02	2.52E-03	0.00
Mitochondrial Electron Transport Chain	15	1	2.16E-03	6.14E+00	2.47E-02	2.52E-03	0.00
Phytanic Acid Peroxisomal Oxidation	19	1	2.16E-03	6.14E+00	2.47E-02	2.52E-03	0.00
Lysine Degradation	20	1	3.94E-03	5.54E+00	2.47E-02	4.31E-03	0.00
Biotin Metabolism	7	1	3.94E-03	5.54E+00	2.47E-02	4.31E-03	0.00
Betaine Metabolism	18	1	9.47E-03	4.66E+00	2.84E-02	1.00E-02	0.00
Transfer of Acetyl Groups into Mitochondria	18	1	3.15E-02	3.46E+00	6.29E-02	3.24E-02	0.07
Cysteine Metabolism	24	1	6.61E-01	4.13E-01	6.61E-01	6.61E-01	0.00

Supplementary Table S3. The perturbed metabolic pathways in the malignant lesions vs chronic thyroiditis. The table was created using Metaboanalyst 4.0 software. https://www.metaboanalyst.ca.

	Total Cmpd	Hits	Raw p	-log(p)	Holm adjust	FDR	Impact
Oxidation of Branched Chain Fatty Acids	22	1	3.42E-10	2.18E+01	1.20E-08	1.64E-09	0.00
Valine, Leucine and Isoleucine Degradation	51	1	3.42E-10	2.18E+01	1.20E-08	1.64E-09	0.00
Ketone Body Metabolism	12	1	3.42E-10	2.18E+01	1.20E-08	1.64E-09	0.00
Butyrate Metabolism	16	1	3.42E-10	2.18E+01	1.20E-08	1.64E-09	0.00
Mitochondrial Electron Transport Chain	15	1	3.42E-10	2.18E+01	1.20E-08	1.64E-09	0.00
Phytanic Acid Peroxisomal Oxidation	19	1	3.42E-10	2.18E+01	1.20E-08	1.64E-09	0.00
Citric Acid Cycle	26	2	3.74E-10	2.17E+01	1.20E-08	1.64E-09	0.21
Warburg Effect	49	2	3.74E-10	2.17E+01	1.20E-08	1.64E-09	0.05
Phosphatidylcholine Biosynthesis	18	2	8.12E-10	2.09E+01	2.19E-08	2.84E-09	0.17
Phospholipid Biosynthesis	25	2	8.12E-10	2.09E+01	2.19E-08	2.84E-09	0.07
Sphingolipid Metabolism	36	2	1.05E-09	2.07E+01	2.62E-08	3.33E-09	0.00
Carnitine Synthesis	16	3	1.59E-09	2.03E+01	3.81E-08	4.63E-09	0.00
Arginine and Proline Metabolism	48	2	2.07E-09	2.00E+01	4.76E-08	5.17E-09	0.00
Glutamate Metabolism	45	2	2.07E-09	2.00E+01	4.76E-08	5.17E-09	0.00
Inositol Metabolism	28	1	5.53E-07	1.44E+01	1.16E-05	1.08E-06	0.15
Galactose Metabolism	31	1	5.53E-07	1.44E+01	1.16E-05	1.08E-06	0.00
Inositol Phosphate Metabolism	22	1	5.53E-07	1.44E+01	1.16E-05	1.08E-06	0.00
Phosphatidylinositol Phosphate Metabolism	14	1	5.53E-07	1.44E+01	1.16E-05	1.08E-06	0.00
Glutathione Metabolism	19	1	8.14E-06	1.17E+01	1.38E-04	1.36E-05	0.06
Purine Metabolism	63	1	8.14E-06	1.17E+01	1.38E-04	1.36E-05	0.00
Alanine Metabolism	14	1	8.14E-06	1.17E+01	1.38E-04	1.36E-05	0.00
Porphyrin Metabolism	36	2	9.26E-06	1.16E+01	1.38E-04	1.47E-05	0.00
Methionine Metabolism	39	3	3.50E-05	1.03E+01	4.55E-04	5.33E-05	0.01
Betaine Metabolism	18	1	3.92E-05	1.01E+01	4.70E-04	5.71E-05	0.00
Phosphatidylethanolamine Biosynthesis	13	2	5.94E-05	9.73E+00	6.53E-04	8.31E-05	0.00
Glycine and Serine Metabolism	50	2	1.48E-04	8.82E+00	1.48E-03	1.92E-04	0.12
Ammonia Recycling	25	2	1.48E-04	8.82E+00	1.48E-03	1.92E-04	0.00
Selenoamino Acid Metabolism	28	1	3.45E-04	7.97E+00	2.76E-03	4.16E-04	0.00
Homocysteine Degradation	7	1	3.45E-04	7.97E+00	2.76E-03	4.16E-04	0.00
Threonine and 2-Oxobutanoate Degradation	13	1	2.22E-03	6.11E+00	1.33E-02	2.60E-03	0.00
Bile Acid Biosynthesis	59	3	7.02E-03	4.96E+00	3.51E-02	7.92E-03	0.00
Lysine Degradation	20	1	2.02E-01	1.60E+00	8.09E-01	2.15E-01	0.00
Biotin Metabolism	7	1	2.02E-01	1.60E+00	8.09E-01	2.15E-01	0.00
Cysteine Metabolism	24	1	4.18E-01	8.73E-01	8.35E-01	4.23E-01	0.00
Transfer of Acetyl Groups into Mitochondria	18	1	4.23E-01	8.61E-01	8.35E-01	4.23E-01	0.07

Supplementary Table S4. The perturbed metabolic pathways in the benign lesions vs. chronic thyroiditis. The table was created using Metaboanalyst 4.0 software. https://www.metaboanalyst.ca.

Metabolic pathway	Malignant lesions Vs Colloid goiter	Benign lesions vs Colloid goiter	Malignant lesions vs Chronic thyroiditis	Benign lesions vs Chronic thyroiditis
**Glycine and Serine metabolism**	47.72	27.81	18.51	9.18
**Phosphatidylcholine bisynthesis**	10.33	40.18	11.08	28.10
**Inositol metabolism**	0.66	5.28	10.44	21.09
**Citric acid cycle**	24.74	36.33	8.74	21.00
**Warburg effect**	55.49	28.96	24.05	10.10

Supplementary Table S5. Fold enrichment values obtained from enrichment analysis for the most important metabolic pathways perturbed in thyroid lesions in reference to non-tumoral tissue.

The table was created using Metaboanalyst 4.0 software. https://www.metaboanalyst.ca.
